# Single-cell transcriptomics reveal distinctive patterns of fibroblast activation in heart failure with preserved ejection fraction

**DOI:** 10.1007/s00395-024-01074-w

**Published:** 2024-09-23

**Authors:** Jan D. Lanzer, Laura M. Wienecke, Ricardo O. Ramirez Flores, Maura M. Zylla, Celina Kley, Niklas Hartmann, Florian Sicklinger, Jobst-Hendrik Schultz, Norbert Frey, Julio Saez-Rodriguez, Florian Leuschner

**Affiliations:** 1 Institute for Computational Biomedicine, Heidelberg University, Im Neuenheimer Feld 130.3, 69120 Heidelberg, Germany; 2 Internal Medicine II, Heidelberg University Hospital, Heidelberg, Germany; 3 Informatics for Life, Heidelberg, Germany; 4 German Center for Cardiovascular Research (DZHK), Partner Site Heidelberg, Heidelberg, Germany; 5 Department of Cardiology, Internal Medicine III, Heidelberg University Hospital, Im Neuenheimer Feld 410, 69120 Heidelberg, Germany

**Keywords:** Heart failure with preserved ejection fraction (HFpEF), Fibroblast activation, Angiopoietin-like 4, Single-cell RNA sequencing, Immune activation

## Abstract

Inflammation, fibrosis and metabolic stress critically promote heart failure with preserved ejection fraction (HFpEF). Exposure to high-fat diet and nitric oxide synthase inhibitor N[w]-nitro-l-arginine methyl ester (L-NAME) recapitulate features of HFpEF in mice. To identify disease-specific traits during adverse remodeling, we profiled interstitial cells in early murine HFpEF using single-cell RNAseq (scRNAseq). Diastolic dysfunction and perivascular fibrosis were accompanied by an activation of cardiac fibroblast and macrophage subsets. Integration of fibroblasts from HFpEF with two murine models for heart failure with reduced ejection fraction (HFrEF) identified a catalog of conserved fibroblast phenotypes across mouse models. Moreover, HFpEF-specific characteristics included induced metabolic, hypoxic and inflammatory transcription factors and pathways, including enhanced expression of Angiopoietin-like 4 (Angptl4) next to basement membrane compounds, such as collagen IV (Col4a1). Fibroblast activation was further dissected into transcriptional and compositional shifts and thereby highly responsive cell states for each HF model were identified. In contrast to HFrEF, where myofibroblast and matrifibrocyte activation were crucial features, we found that these cell states played a subsidiary role in early HFpEF. These disease-specific fibroblast signatures were corroborated in human myocardial bulk transcriptomes. Furthermore, we identified a potential cross-talk between macrophages and fibroblasts via SPP1 and TNFɑ with estimated fibroblast target genes including Col4a1 and Angptl4. Treatment with recombinant ANGPTL4 ameliorated the murine HFpEF phenotype and diastolic dysfunction by reducing collagen IV deposition from fibroblasts in vivo and in vitro. In line, ANGPTL4, was elevated in plasma samples of HFpEF patients and particularly high levels associated with a preserved global-longitudinal strain. Taken together, our study provides a comprehensive characterization of molecular fibroblast activation patterns in murine HFpEF, as well as the identification of Angiopoietin-like 4 as central mechanistic regulator with protective effects.

**Supplementary Information:**

The online version contains supplementary material available at 10.1007/s00395-024-01074-w.

## Introduction

Heart failure with preserved ejection fraction (HFpEF) represents one of the largest unmet clinical needs in cardiovascular medicine, given that it accounts for about 50% of heart failure (HF) patients and is increasing in prevalence [63]. However, apart from SGLT2 inhibitors, no effective treatment strategies exist to reduce the associated diastolic dysfunction, fibrosis, hypertrophy and the resulting pronounced morbidity and mortality. Therapeutic concepts and established drugs for the treatment of heart failure with reduced ejection fraction (HFrEF) failed broadly when tested for beneficial effects in HFpEF, suggesting fundamentally different pathomechanisms [44, 63].

HFpEF comprises a complex and multifactorial interplay of the disease promoting risk factors, such as hypertension, obesity, metabolic syndrome, chronic inflammation, and aging. Suitable animal models were missing until a few years ago, when a two-hit mouse model combining a 60% high-fat diet with inhibition of the constitutive nitric oxide synthase by Nω-nitro-l-arginine methyl ester (L-NAME) recapitulated metabolic and hypertensive stress in HFpEF [84, 103]. Analysis of this model led to major mechanistic insights in the pathophysiology of hypertrophy and cardiac immunometabolic alterations in HFpEF [83, 84, 100] and potential therapeutic targets. Since these studies focused predominantly on cardiomyocyte hypertrophy and metabolism [63], little knowledge was gathered about the distinct role of cardiac interstitial cells and their cross-talk in ventricular stiffening and fibrosis [63, 83].

Single-cell RNA sequencing (scRNAseq) allows for the quantification of transcriptional changes of individual cells and description of cell phenotype heterogeneity. Consequently, scRNAseq has opened the door for fundamental insights into cellular heterogeneity, developmental biology and molecular disease processes in the cardiovascular field [2, 31, 50]. Thus, its application to a HFpEF model could shed light on the cellular disease mechanisms.

Here we present to our knowledge the first scRNAseq analysis of the ventricular interstitium in mice receiving L-NAME and high-fat diet (further called HFpEF model) in early stages of diastolic dysfunction. We compared fibroblast phenotypes and disease signatures by integration with scRNAseq data from other HF models that recapitulate HFrEF and identified HFpEF-specific patterns of fibroblast activation. We characterized HFpEF-associated fibrotic signatures and compared them with human bulk references, providing new pathophysiological hypotheses relevant for the understanding of fibrosis in HFpEF necessary for future anti-fibrotic drug development.

## Results

### Disease model and data description

**Fig. 1:**
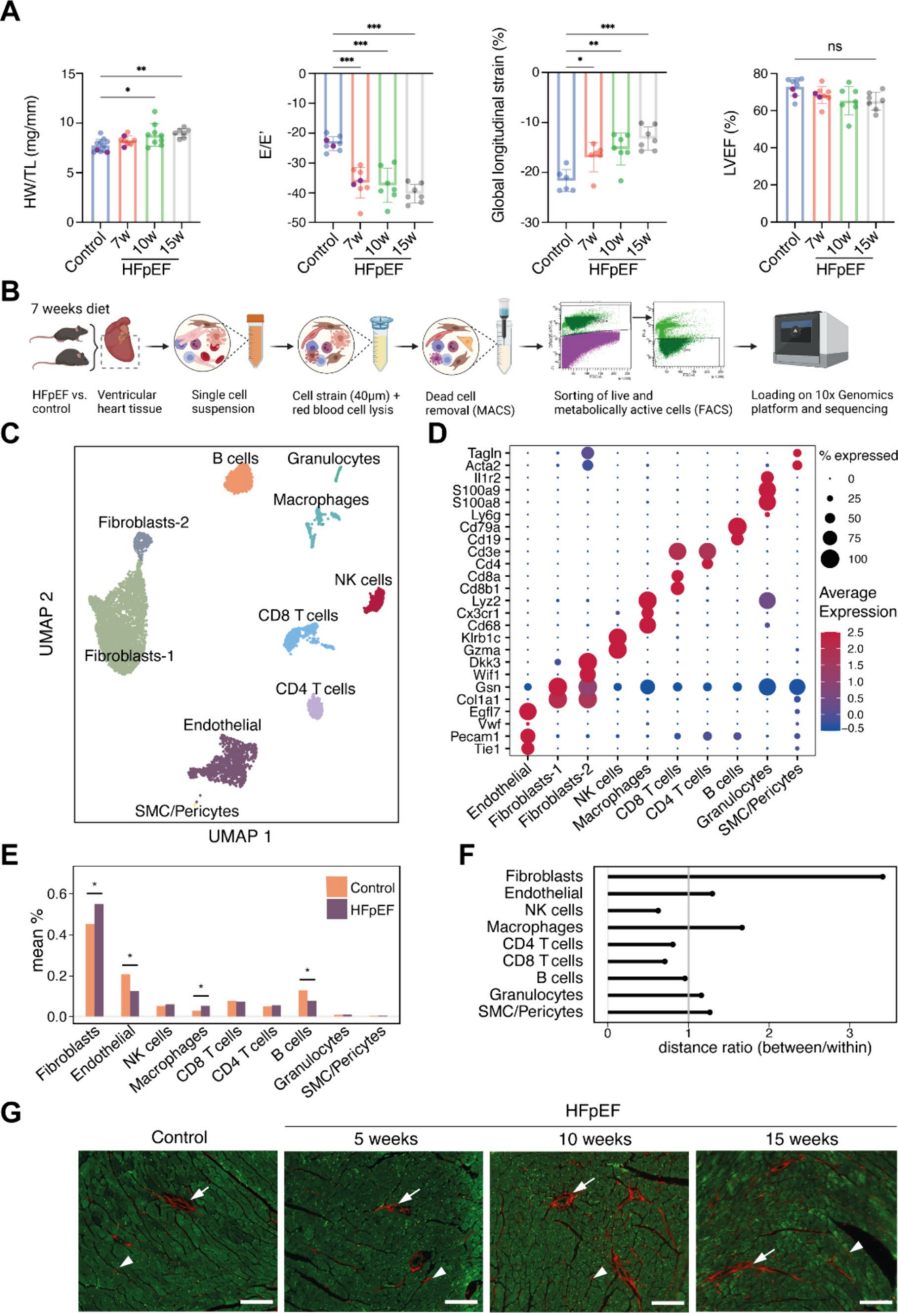
Study model and cell type assignment. **A** Murine HFpEF model characterization by ratio of heart weight to tibia length (HW/TL) and echocardiographic hallmarks (E/E’, global-longitudinal strain and LVEF), purple data points represent the animals used for single-cell RNA sequencing (scRNAseq). Statistical analysis performed by one-way ANOVA, bar graphs indicate mean ± SD, *p < 0.05, **p < 0.01, ***p < 0.001. ns = deemed not significant (*p* > 0.05), LVEF = left ventricular ejection fraction, *w* weeks. **B** Schematic summary of experimental setup for scRNAseq experiments using mice after 7 weeks of HFpEF or control diet. Created with BioRender.com. **C** UMAP embeddings of normalized scRNAseq data after processing and filtering. **D** Marker gene expression for cell type assignment. **E** Cell type composition of main cell types as mean percentage per group, compared between HFpEF and control mice. *p < 0.05, *p* values were calculated via label permutation. **F** Cosine distance ratios of highly variable genes between pseudobulked cell type profiles. Median between group distance is divided by median within group distance. **G** Representative Picrosirius-Red stainings of interstitial fibrotic fibers (arrowheads) and perivascular fibrosis (arrows) from control and different stages of HFpEF heart sections. Imaging performed in 594 nm (Picrosirius-Red) and 488 nm (autofluorescence) channels. White scale bars in the right bottom corner correspond to 100 μm

To mimic HFpEF, we used the established two-hit mouse model that induces metabolic and hypertensive stress by 60% high-fat diet and L-NAME, respectively [84]. From 7 weeks of dietary intervention onwards, a diastolic dysfunction phenotype was observed echocardiographically under preservation of systolic left ventricular function (Fig. 1A, Supp. Fig. 1). Body and heart weight, normalized to tibia length, increased concordantly indicating obesity and cardiac hypertrophy (Fig. 1A, Supp. Fig. 1A–C). To describe this early remodeling, we isolated cardiac interstitial cells after 7 weeks by MACS^Ⓡ^ dead cell depletion and FACS sorting of live and metabolically active cells (Fig. 1B). We performed scRNAseq with the 10 × Chromium droplet based platform to analyze cellular transcriptomic changes within cardiac ventricular interstitial cells of two control and two HFpEF murine hearts. After processing and quality control we retained expression profiles of 6,132 cells described by 15,046 genes (mean UMI coverage per cell: 2,838) (Supp. Fig. 2). Unsupervised clustering yielded 10 distinct clusters (Fig. 1C) representing major cell types of the cardiac interstitium based on their top marker genes and known canonical markers. We identified two fibroblast clusters (Col1a1 + and Wif1 +), endothelial cells (EC) (Pecam1 +), natural killer cells (Gzma +), macrophages (CD68 +), T effector cells (CD8 +) and T helper cells (CD4 +), B cells (CD19 +), granulocytes (S100a9 +), smooth muscle cells and pericytes (Acta2 +) (Fig. 1D).

### Cell type composition and molecular profiles suggest fibroblast and macrophage involvement in cardiac remodeling

To identify interstitial cells involved in HFpEF remodeling, we first compared the cellular composition in control with HFpEF cardiac tissue, and evaluated the significance of compositional changes by label permutation (see methods). This yielded a modest increase of fibroblasts and macrophages and decrease of B cells and ECs in HFpEF (Fig. 1E, Supp. Fig. 3A, B).

As cell type compositions are not independent and therefore only partially informative of the importance of a cell type for disease process, we assessed whether the variation of gene expression between experimental groups was higher than the variability expected within a single group (see methods) (Fig. 1F). We found that fibroblasts displayed the highest ratio of ‘between to within group distance’ followed by macrophages. We applied a cell type prioritization method to rank cell types by classifier performance. This classifier was trained to separate healthy from diseased cells and can provide an additional estimate for magnitude of molecular changes in cell types [89]. This yielded the highest performance for macrophages and ECs, followed by modest performance for fibroblasts (Supp. Fig. 3C). L-NAME treatment directly targets ECs, expected to induce direct transcriptional changes. Taken together, the compositional change and molecular differences suggested that fibroblasts and macrophages could constitute important contributors to the early HFpEF-associated remodeling.

Fibroblast activity relates to cardiac fibrosis, which is a hallmark feature of human HFpEF [92]. In parallel, we found a qualitative increase of interstitial and perivascular collagen deposition with time in the HFpEF model (Fig. 1G, Supp. Fig. 1M). Thus, 7 weeks of HFpEF diet already recapitulated hallmark features of HFpEF including cardiac fibrosis and mild functional changes of the left ventricle at this time point. While fibrosis represents one of the major pathomechanisms without current mitigating therapeutic options, investigating early fibroblast activation is of high interest to understand HFpEF-related cardiac fibrosis.

### Fibroblast phenotype definitions across murine heart failure models

**Fig. 2:**
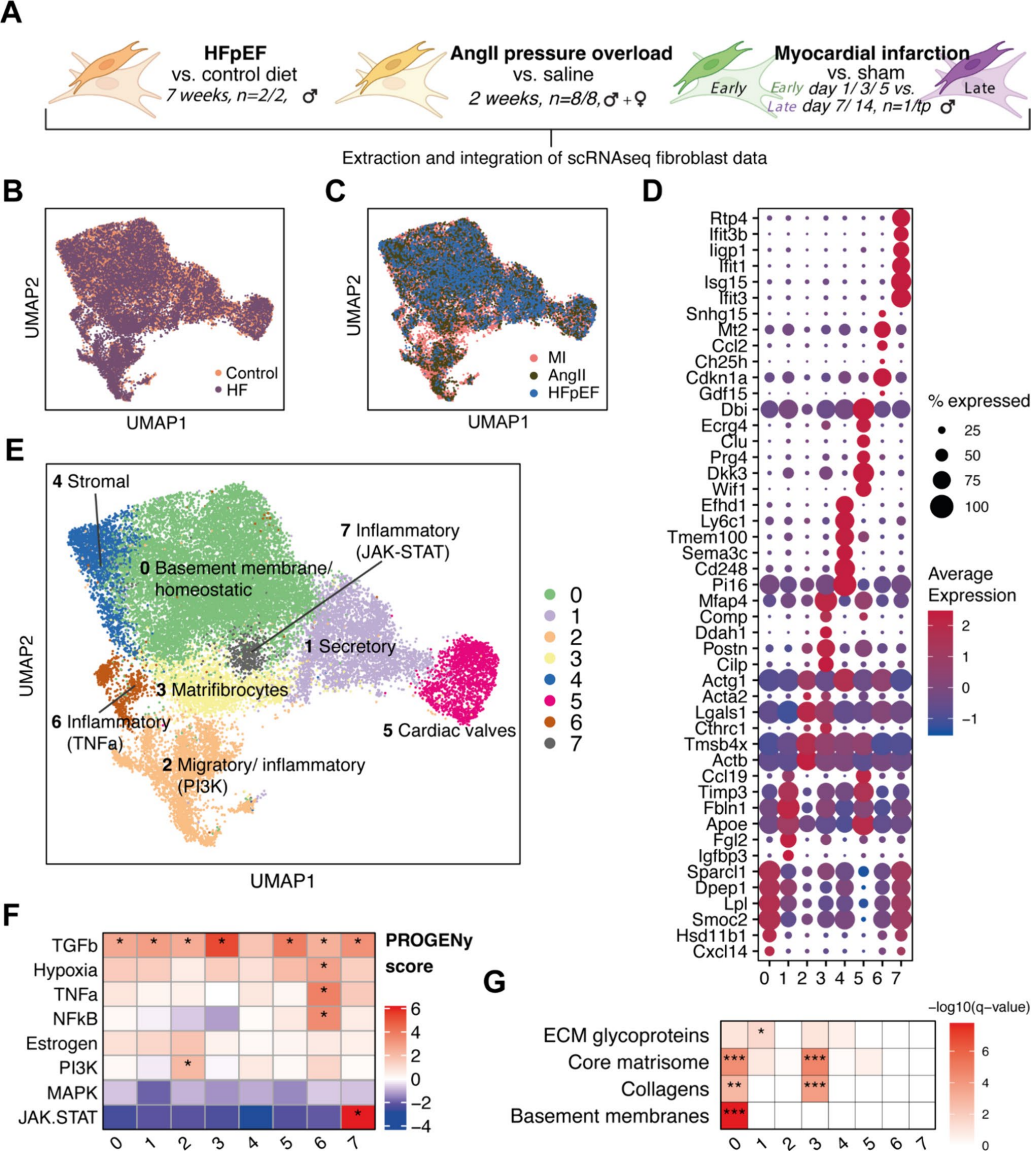
Integrated atlas of cardiac fibroblasts from different disease models. **A** Schematic of the integrated murine HFpEF and HFrEF (AngII and MI) fibroblast studies. **B** + **C** UMAP embeddings of integrated fibroblasts, colored by disease (HF, Heart Failure) vs. control (**B**), study (**C**). **D** Overview of top cell state marker expression of integrated fibroblast states. **E** UMAP embeddings, showing the integrated fibroblast atlas colored by cell clusters, i.e. the integrated fibroblast states (IFS). Labels indicate possible fibroblast differentiations based on functional characterization. **F** Estimated pathway activities with PROGENy based on effect size (avg log2 fold change) of footprint genes in integrated fibroblast states. *PROGENy *z* score > 2. **G** Overrepresentation analysis of extracellular matrix related gene sets with markers of integrated fibroblast states. Hypergeometric test with Benjamini–Hochberg correction, *q < 0.05, **q < 0.01, ***q < 0.001

Cardiac fibroblasts accomplish a wide range of biological functions, crucial for tissue homeostasis and architecture [34]. In human HFrEF and HFpEF, cardiac fibrosis represents a major axis of reparative and adverse remodeling. While histologically HFpEF has been associated with interstitial and perivascular fibrosis, the underlying functional characteristics of fibroblast activation remain unknown [70, 92]. Thus, we sought to compare HFpEF fibroblast activation with other cardiac fibrotic disease etiologies by integration of our single-cell data with two other single-cell resources that represent different types of HFrEF: first, a model for cardiac fibrosis and hypertrophy by hypertensive stress induced by 2 weeks of angiotensin II (AngII) administration [62] and second, an acute myocardial infarction (MI) model [31] that assessed early (< day 7) and later ischemic remodeling (day 7–14) (Fig. 2A). The MI model is characterized by cell death and associated replacement fibrosis [33] while the AngII administration causes initially extensive reactive fibrosis [85].

We uniformly processed studies, annotated cell types and identified fibroblasts by selecting Col1a1^+^ , Pdgfra^+^ and Gsn^+^ cells (Supp. Fig. 4). Fibroblasts from three datasets were then integrated with *Harmony *[51] while accounting for sample and study batch effects that resulted in an integrated cardiac fibroblast atlas of 26,455 cells, capturing a wide spectrum of phenotype diversity across HF models. Study and sample batch effects were satisfactorily mitigated (see methods) (Fig. 2B, C).

Previous studies identified cardiac fibroblast phenotypes at the single-cell level in healthy and diseased hearts with limited consistency [15, 29, 31, 43, 62, 80]. Thus, the integration allowed us to robustly define high-level fibroblast phenotypes across different cardiac remodeling scenarios that enable direct model comparison. In the integrated atlas, we identified eight integrated fibroblast cell states (IFS) by performing unsupervised clustering. Each study contributed to all IFS (Supp. Fig. 5A). To functionally characterize the IFS, we derived state markers via differential expression analysis (Fig. 2D, Supp. Fig. 5B, Supp. Table 1).

First, we aimed to identify which of these IFS constitute cardiac specific fibroblasts. For this, we compared IFS markers with markers of fibroblast states identified in a cross organ fibroblast atlas (Supp. Fig. 5C) [15]. IFS 0 (Col15a1^+^), 3 (Comp^+^) and 4 (Pi16^+^) displayed high marker overlap (hypergeometric test *p* < 0.01) which suggested that these states might represent fibroblast phenotypes shared across organs. Conversely, IFS 1, 2, 5, 6 and 7 displayed weaker or ambiguous associations and could represent rather cardiac-specific fibroblast phenotypes.

We expected that IFS could represent functional niches (i.e. specialization of fibroblasts to fulfill certain tissue functions). We characterized these functional niches (Fig. 2E) by performing pathway activity (Fig. 2F) and gene set enrichment analysis (Fig. 2G, Supp. Fig. 5F). **IFS 0** fibroblasts were the most abundant cell type in every dataset (Supp. Fig. 5D) and have been described as homeostatic fibroblasts that are characterized by Col15a1 and Dpep1 expression [15]. **IFS 4** fibroblasts were characterized by Pi16 expression and constitute adventitial stromal cells that might accomplish a reservoir function for downstream fibroblast differentiation [15, 26]. The **IFS 3** can be termed matrifibrocytes and are characterized by Cilp, Thbs4, Comp and Postn expression [29, 62]. Pathway analysis indicated that IFS 3 demonstrated highest TGFβ activity (Fig. 2F), which highlighted the pro-fibrotic potential of this IFS. Extracellular matrix (ECM) remodeling is a major operation of fibroblasts and was assessed by enrichment of ECM related gene sets [66], suggesting that IFS 0 and IFS 3 fibroblasts were the main ECM producers: both were characterized by expression of collagens and core matrisome-related genes, while IFS 0 uniquely expressed genes associated with the basement membrane (e.g. Col4a1, Lamb1, Hspg2, Col15a1)(Fig. 2G). We identified three IFS with inflammatory profiles: IFS 2, IFS 6 and IFS 7. **IFS 2** appeared to be a heterogenous group of fibroblasts that were partly characterized by Acta2 and Actb expression which constitute myofibroblast characteristics, as well as pro-inflammatory genes involved in antigen processing and representation (Psmd8, Psma6, Vamp8) and Chaperonin containing T-complex polypeptide genes (CCT3, CCT7, CCT4, CCT8) that have been associated with proliferative and fibrotic tissue remodeling [8, 74, 106]. Furthermore, IFS 2 exhibited highest PI3K pathway activity which has been shown to enable fibroblast migration [69, 102]. **IFS 6** fibroblasts were characterized by pro-inflammatory NFκB and TNFα signaling (Fig. 2F) and cytokine expression of Ccl2, Cxcl5 suggested that IFS 6 participates in immune cell attraction. **IFS 7** cells formed a small cluster that every study contributed to with a comparatively small number of cells (Supp. Fig. 5D) and was characterized by JAK-STAT activity and interferon-γ-related gene expressions (Ifit3, Isg15). The JAK–STAT pathway has been linked to fibroblast activity in rheumatoid arthritis [27, 47] and osteoporosis [104] but its function in cardiac fibroblasts remains unclear. **IFS 5** was characterized by, among others, Wif1 and Dkk3 expression. In the heart, Wif1^+^ cells were previously shown to localize at the cardiac valves and their adjacent hinge regions [65], however, with unknown functionality. **IFS 1** was characterized by expression of typically secreted gene products including insulin-like growth factor 1 (Igf1) and fibrinogen-like protein 2 (Fgl2), which can control cardiomyocyte growth [28, 93], next to the Igf-function regulators insulin-like growth factor-binding proteins (Igfbp3, Igfbp4) [32], glycoproteins like fibulin-1 (Fbln1), extracellular matrix protein 1 (Ecm1) and matrix-gla protein (Mgp).

In summary, the phenotype atlas of murine cardiac fibroblasts can be broadly categorized as a set of eight fibroblast states, characterized by distinct key molecular programmes including ECM remodeling (IFS 0, 3), immune modulation (IFS 2,6,7), secretion (IFS 1), and presumably tissue homeostasis (IFS 1, 5).

### Distinct fibroblast signature of HFpEF

**Fig. 3:**
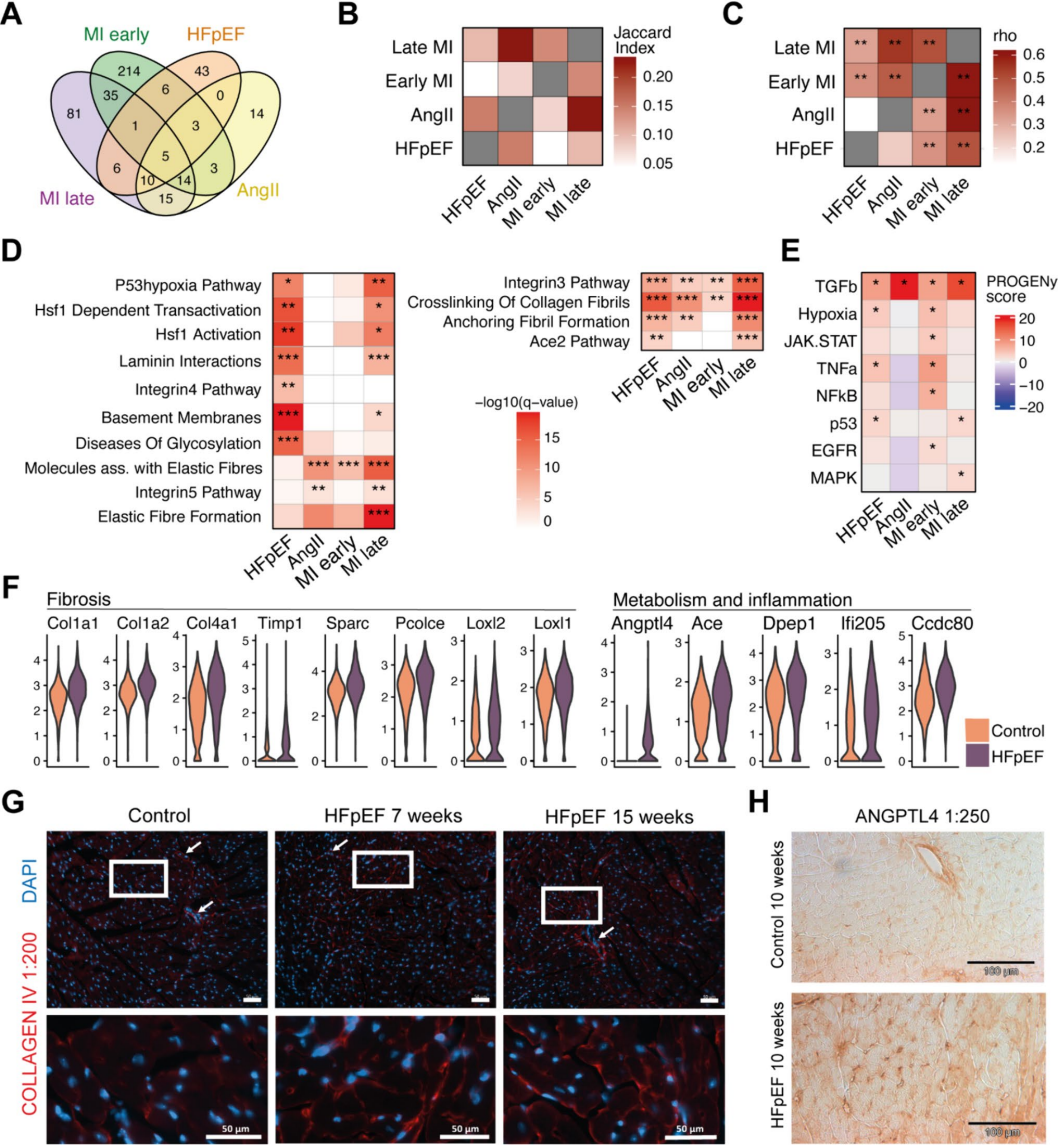
Comparison and interpretation of fibroblast disease signatures from different heart failure models. **A** Comparing intersections of upregulated genes in different heart failure (HF) models. **B** Intersection quantification via Jaccard index. **C** Comparison of direction of regulation between studies. Pearson correlation was calculated between log fold change vectors of signature genes in pairwise comparisons. Each study comparison was based on the upregulated genes from the study on the x-axis. **p < 0.01. **D** Heatmaps of gene set overrepresentation in study specific fibroblast disease signatures. Hypergeometric test with Benjamini–Hochberg correction, *q < 0.01, **q < 0.001, ***q < 0.0001. **E** Estimated pathway activities with PROGENy based on effect size (log fold change) of footprint genes compared between HF models. **F** Expression values of selected fibrosis and inflammatory genes in individual fibroblasts in HFpEF (purple) and control (orange) mice. All genes were significantly upregulated (Wilcoxon test, adj. *p* value < 0.05). **G** Immunofluorescence images of collagen IV (red) and DAPI (blue) staining of left ventricular heart sections. Lower panels show magnifications of the areas marked by white boxes. White arrows indicate capillaries or larger blood vessels. Scale bars in the right bottom corner indicate 50 μm length. **H** Immunohistological staining of Angptl4 protein in left ventricular heart sections

To functionally compare fibroblast activation between study models, we performed differential gene expression analysis between control and disease fibroblasts for each study independently to avoid cross study batch comparison. Since the MI study included multiple timepoints, we separated the samples by calculating signatures of early (days 1, 3, and 5) and later (days 7 and 14) remodeling. The resulting signatures contained a small set of upregulated (Timp1, Col1a1, Col1a2, Loxl1 and Sparc) and downregulated genes common to all disease models (Fig. 3A, Supp. Table 2). Between the HF models we found little overlap regarding the respective differentially expressed genes, except for AngII and late MI signatures (Fig. 3B). Since only a few genes were shared between disease signatures, we asked whether the direction of gene expression regulation, determining whether a gene's activity is increased (upregulated) or decreased (downregulated), is nevertheless consistent between HF models. We correlated fold change regulation of disease signatures between studies and found the strongest correlation between AngII and late MI fibroblasts. Interestingly, the HFpEF signature did not correlate with AngII while displaying moderate agreements with early and late MI, indicating disease-specific fibroblast activation patterns (Fig. 3C).

To further elucidate the differences of these fibrotic features, we characterized signatures by enriching annotated gene sets from the MSig database [17]. Fibrosis signatures across models contained major ECM related gene sets (Supp. Fig. 6A), indicative of a common profibrotic task. The HFpEF signature was uniquely characterized by heat-shock factors, protein glycosylations, basement membrane and laminin components, but did not contain components related to elastic fibers, unlike signatures from AngII and MI models (Fig. 3D). Next, we used fold change regulation of regulon genes to infer upstream transcription factor (TF) activities (Supp. Fig. 6B). Among others, Hsf1, Ppar-ɑ and Ppar-ɣ were suggested to be relevant TFs specifically in HFpEF fibroblasts and could constitute important mediators of metabolic stress response. In addition, Hif1ɑ activity was found in HFpEF and, as expected, in early MI fibroblasts. In HFpEF, hypoxia may occur in obesity-related tissue stress [101], but its impact on cardiac fibroblast function in HFpEF is unknown. All models displayed high Smad3 activity, which is known to be an important driver of cardiac fibrosis via TGFβ signaling [59]. Indeed, when comparing pathway activities (Fig. 3E), TGFβ was active in all HF models, however, strongest activity was found in AngII and late MI models while in early MI fibroblasts proinflammatory TNFα, NFκB, as well as hypoxia, and JAK-STAT pathways were induced. In HFpEF, besides TGFβ, the hypoxia [1, 64], TNFα [79] and p53 [20] pathways were predicted to be activated in fibroblasts.

Besides the upregulation of main ECM components in HFpEF (e.g. Col1a1, Col1a2, Col4a1, Sparc, Pcolce), we found collagen cross linking enzymes (Loxl1, Loxl2), metabolic and inflammation related genes to be induced (e.g. Angptl4, Ace, Dpep1, If205 and Ccd80) (Fig. 3F). Col4a1 is an important component of the basement membrane and its accumulation over time in the HFpEF model was confirmed by immunofluorescence stainings and indicated a collagen IV pattern of interstitial sheathing of cardiac cells (Fig. 3G). Angiopoietin-like 4 (Angptl4) is a lipoprotein lipase inhibitor that was barely expressed in control fibroblasts, but strongly induced in HFpEF. It is known to be regulated via Ppar-ɑ and Ppar-ɣ in other contexts [58]. Hence, Angptl4 could constitute an important indicator of metabolic stress in fibroblasts in HFpEF. Qualitative protein staining of Angptl4 confirmed upregulation especially in the cardiac interstitium (Fig. 3H).

While the common gene expression patterns between HF models related to TGFβ and Smad3 activity together with upregulation of ECM genes, distinctive HFpEF fibroblast activation patterns included upregulation of Angptl4 and other markers of metabolic stress, basement membrane genes, and activation of proinflammatory pathways and TFs.

### Compositional and transcriptional shifts in cardiac fibroblasts

In the previous sections, we characterized integrated fibroblast states (IFS) and interpreted model-specific disease signatures. To combine both perspectives, we investigated how fibroblasts from different IFS contributed to the model-specific cardiac remodeling. This could help us to understand the division of labor between fibroblast states and compare study models from a cell population perspective.

**Fig. 4:**
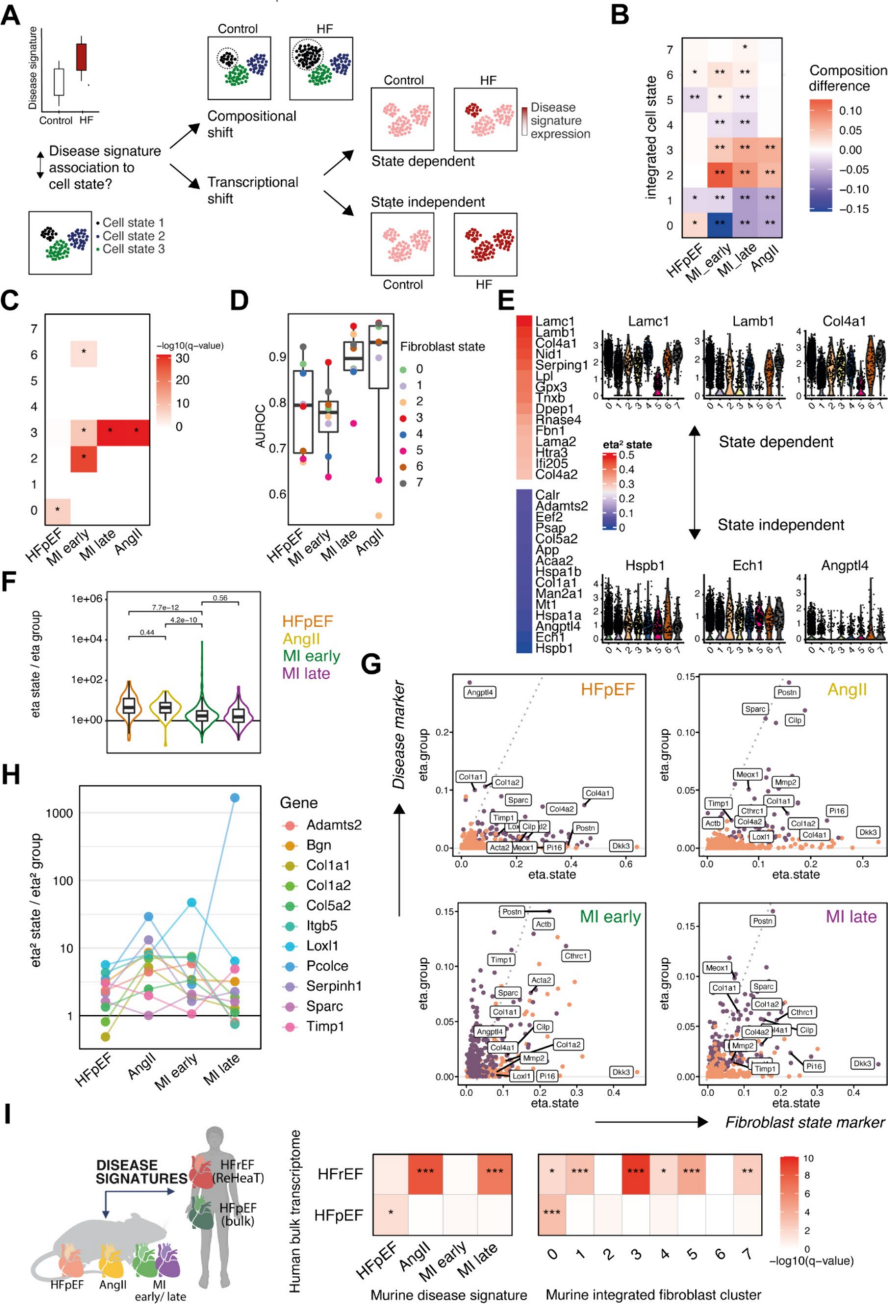
Decomposing Fibroblast disease signatures. **A** Schematic of different expression patterns in regard to cell states that could yield an upregulation of a disease signature. Compositional shifts by expanding cell number are distinguished from transcriptional shifts via uniform (state independent) or non-uniform (state dependent) upregulation of disease signatures. **B** Composition change of integrated fibroblast states (IFS) between control and heart failure per study. *p* values calculated via label permutation, *p < 0.05, **p < 0.01. **C** Overrepresentation analysis of disease-specific fibroblast signatures (*x*-axis) and top 100 IFS markers (*y*-axis). Hypergeometric test, *p < 0.05. **D** Gene set scores of study specific signatures (x-axis) were used to calculate the area under the receiver operator curve (AUROC, *y*-axis) between control and diseased cells within each IFS (color). **E** HFpEF signature expression dependent on IFS category by calculating the explained variance (eta^2^ values) of gene-wise ANOVAs. Violin plots display normalized expression values of three genes with lowest (lower panel) and highest (upper panel) variance explained by cell state. F) Quantification of differences in state-dependent regulation of disease signatures across heart failure models. The ratio of the explained variance by IFS and disease class was calculated for each HF model and its disease signature. Wilcoxon test *p* values are shown. **G**) Explained variance (eta^2^ values) by IFS on x-axis and explained variance by disease class (gene ~ disease class) on y-axis. Violet dots are part of the disease signature. **H** The ratio of explained variance by state and disease class of selected genes that were upregulated in all HF models. **I** Corroboration of murine fibroblast signatures in human myocardial samples. Human HFpEF and HFrEF studies were curated and top differentially upregulated genes were selected (y-axis). Gene set overlaps with fibroblast disease signatures from different study models (left panel) or fibroblast state marker (right panel) (hypergeometric test). *AngII* =angiotensin II model, *HFpEF* heart failure with preserved ejection fraction, *MI* myocardial infarction. *q* value = Benjamini–Hochberg-corrected *p* value, *q < 0.05, **q < 0.01, ***q < 0.001

We conceptualized different patterns of gene expression with respect to cell states that lead to an upregulation of a disease signature (Fig. 4A). First, we distinguished between compositional and transcriptional shifts [71]. The former describes a relatively stable expression within a cell state where the disease signature upregulation is caused by an increase in the proportion of that state. On the other hand, a transcriptional shift constitutes an upregulation without a compositional increase. Here, we propose to differentiate between upregulation focused within a state (state-dependent) and within many or all states (state-independent). We will use these terms to broadly describe gene expression patterns from different HF models; however, we acknowledge that these categories are not exclusive.

To investigate compositional shifts, we calculated compositional changes of IFS between control and diseased mice per study (Fig. 4B). In HFpEF, composition changes were modest and only IFS 0 and 6 expanded slightly (label permutation *p* value < 0.05), while in early MI the highest compositional dynamics were observed with expansion of IFS 2, 3, 5 and 6. Late MI remodeling displayed similar characteristics as the AngII model with an increase of IFS 3 and 2. To support that these compositional shifts were associated with the disease signatures we assessed the overlap of genes between IFS markers and disease signatures (Fig. 4C). We found that IFS 0 shared markers with the HFpEF signature while IFS 3 with AngII, late and early MI signatures; IFS 2 and 6 with early MI signature only (hypergeometric test, *p* < 0.05). This suggested that a compositional shift was happening in all mouse models, but with different emphases of IFS across the HF models. No other model shared the importance of IFS 0 with HFpEF, which could possibly be a unique feature of HFpEF fibrosis.

To investigate transcriptional shifts, we quantified how well fibroblasts within the same IFS could be distinguished regarding their control and disease label as a metric for a transcriptional shift (Fig. 4D, Supp. Fig. 6C, D) (see methods). In general, the signatures were increasingly expressed across IFS and thus a transcriptional shift was apparent in every study model (Area under the receiver operator curves, AUROCs > 0.5). The highest differences were achieved in the MI (early and late) models compared to HFpEF and AngII models which could indicate that the latter displayed a less pronounced transcriptional shift. This might be explained by acute tissue injury after MI as opposed to the chronic stimuli of AngII administration or HFpEF diet. In addition, a different hierarchy of IFS responsiveness was observed, indicating that the transcriptional shift is partially state dependent: While the highest transcriptional shifts in HFpEF were found in IFS 7 and 0, the other models consistently displayed IFS 3 and 7 as the most responsive states (Fig. 4D). Furthermore, IFS 5 fibroblasts were poorly responsive in all study models, suggesting them to be less relevant for disease remodeling, which may be related to their suggested localization at the cardiac valves.

In summary, we found that fibroblasts from IFS that did not display compositional shifts, nevertheless, contributed to the remodeling by upregulating respective disease signatures. However, fibroblasts from IFS that do display compositional shifts displayed also a high transcriptional shift, suggesting that both concepts are biologically closely related. We concluded that IFS that displayed (i) state marker overlap with the disease signature, (ii) compositional increase and (iii) a high within-state-transcriptional-shift, represented the desired functional niches and thereby the prioritized states in each HF model. Those states were IFS 0 in HFpEF, IFS 2, 3 and 6 in early MI and IFS 3 in late MI and AngII. However, besides this prioritization, all states apart from IFS 5 partook in cardiac remodeling.

### Decomposing disease signatures and state dependency

After characterizing transcriptional and compositional shifts in the HF models, we next aimed to decompose disease signatures regarding their state dependency. To quantify this dependency we calculated eta^2^ values (see methods). In HFpEF fibroblasts, genes related to the basement membrane (Lamc1, Lamb1, Col4a1, Nid1) yielded highest eta^2^ values suggesting a state-dependent expression. Metabolism (Angptl4, Ech1, Man2a, Acaa2) and fibrosis associated genes (Col1a1, Col1a2, Timp1, Mmp1) displayed rather state-independent expression patterns (Fig. 4E). This indicated that basement membrane remodeling might be a functional specialization of fibroblasts, while the upregulation of metabolic and protein stress together with non-basement membrane ECM markers were a common gene program of fibroblasts in HFpEF.

To compare these expression patterns between HF models, we calculated eta^2^ values for state (indicative of state dependency) and group labels (indicative of an upregulation) for all HF models separately (see methods).

First, we quantified the ratio of both eta^2^ values to compare the general state dependency of disease signatures between HF models (Fig. 4G). The two chronic models (HFpEF & AngII) displayed a more state-dependent transcriptional shift compared to the MI (late & early) fibroblasts (Wilcoxon test, *p* < e10), suggesting that the state-dependent fibroblast response might be a characteristic of chronic remodeling.

Second, we compared the state dependency of single genes between HF models (Fig. 4H). State markers like Dkk3 (IFS 5) or Pi16 (IFS 4) displayed high state dependency and low disease dependency in all HF models, serving as examples for genes that are state markers but without disease involvement. Angptl4 was exposed as a state-independent marker, specific for HFpEF. Postn displayed high group and state related variance in all HF models except HFpEF, and thus represented a crucial marker with high disease association and state dependency. Next, we focused on the core intersection of upregulated genes in all HF models and assessed whether their regulation regarding state dependency might differ between HF models (Fig. 4H). We found that most genes were regulated state dependently, consistent across models. However, Col1a1 and Col1a2 were expressed state dependently in all HF models, except HFpEF (Fig. 4G). Collagen I is a main ECM component and crucial for integrity and stiffness of fibrotic tissue and it has been reported that matrifibrocytes in the heart are responsible for the deposition of collagen I [49]. Our findings could indicate that collagen I deposition in the early HFpEF model might not be related to a state-dependent task since matrifibrocytes were not activated yet and the early fibrosis was achieved by a joint collagen production by fibroblasts of all phenotypes.

To demonstrate how the transcriptional shifts of the discussed key genes relate to IFS, we quantified within state regulation of single genes (AUROCs) (Supp. Fig. 6E) and found that Col1a1 and Col1a2 were upregulated in almost all IFS across models, showing highest upregulation within IFS 3 in non-HFpEF models. Col4a1 and Col4a2, although state dependent expressed in all models, displayed a high transcriptional shift in most IFS in HFpEF. This further elucidates that genes that are state dependently expressed between fibroblasts (such as collagen IV in IFS 0 or collagen I in IFS 3) were also upregulated by other IFS but only in the respective disease context. In addition, Angptl4 displayed low state-dependent variance and a high transcriptional shift in all IFS in HFpEF, possibly rendering it a key marker of state-independent metabolic fibroblast stress.

Differential gene expression analysis can be confounded by background gene expression in single-cell transcriptomics that could be associated with increased cell dissociation in diseased tissue affecting contrast comparison. Other cell types in our single-cell data could not be separated on the basis of the discussed disease signatures, suggesting that the discussed transcriptional shifts were not confounded by background expression (Supp. Fig. 6F). Furthermore, the low correlation between HFpEF and other HF signatures (from Fig. 3B, C) caused other disease signatures to fail separating HFpEF fibroblasts from control.

### Corroborating fibroblast signatures in humans and mice

In the previous section, we established the IFS prioritization by the different HF models. To explore whether this IFS to HF phenotype association could be recovered in humans, we curated myocardial bulk transcriptomic signatures acquired from HFrEF and HFpEF patients. For HFrEF, we relied on a meta-analysis of a total of 653 patients with end-stage heart failure [75]. For HFpEF, limited by data availability, we re-analyzed data from 5 patients that underwent coronary artery bypass graft surgery and met the echocardiographic and diagnostic criteria for HFpEF [22]. We selected top upregulated genes from both bulk resources and performed overrepresentation analysis with the murine fibroblast disease signatures (Fig. 4I, left panel). The murine AngII and late MI signatures displayed a significant overlap with the human HFrEF bulk reference, while murine HFpEF signatures were enriched in the human HFpEF bulk reference (hypergeometric test, p < 0.05). Next, we addressed whether this intersection of disease signals between mouse and human could also be recovered for IFS markers (Fig. 4I, right panel). We found that markers for the IFS 3 (matrifibrocytes) were overrepresented in the human HFrEF signature, in agreement with recent reports from human single-cell studies [18, 76]. In the human HFpEF signature, only IFS 0 state markers were overrepresented. This could possibly suggest the relevance of IFS 0 and its functional niche for human HFpEF. In general, the presented fibroblast signatures from AngII, MI and HFpEF, as well as the IFS prioritizations of models are partially conserved across species (mouse to human) as well as across data modalities (single-cell to bulk RNAseq).

Matrifibrocyte activation is a crucial event in cardiac fibrosis [35] and we further explored the role of this event in the murine HFpEF model. First, we compared the protein expression of myofibroblast and matrifibrocyte markers in HFpEF with an MI model (Supp. Fig. 7A). The matrifibrocyte marker Cilp (Cartilage Intermediate Layer Protein) is an ECM protein abundant in articular cartilage and has been implicated in cardiac fibrosis before [42, 67]. Immunohistological staining of Cilp displayed moderate perivascular protein expression in myocardial tissue of 10 weeks HFpEF compared to control mice, while strong expression was observed after MI (Supp. Fig. 7B). Fibroblast activation protein (FAP) was introduced as a marker for myofibroblast activation in cardiac fibrosis[81, 97]. FAP stainings demonstrated that its expression could only be observed in the MI fibrotic zone (Supp. Fig. 7B). We further assessed FAP expression within the whole murine organism after 15 weeks of HFpEF diet by PET–CT based^68^ Ga-FAPi-46 uptake (Supp. Fig. 7C), yielding no relevant expression patterns across organs. This data indicated that based on myofibroblast and matrifibrocyte markers (Fap and Cilp, respectively) the HFpEF model was not associated with a strong response of these cell states.

Second, we explored myocardial gene expression after 10 and 15 weeks of HFpEF diet (compared to 7 weeks in the single-cell data) (Supp. Fig. 8A). We contrasted both timepoints to control mice and found that the HFpEF, MI and AngII disease signatures all enriched at 10 and 15 weeks (linear regression, p < 0.05, Supp. Fig. 8C). When comparing IFS markers, we found that IFS 0, 1, 3, 5 and 7 enriched significantly (linear regression, *p* < 0.05) with IFS 3 yielding the highest enrichment score (Supp. Fig. 8D). This suggested that a matrifibrocyte activation as reflected by IFS 3 marker upregulation might have occurred later than 7 weeks in the HFpEF mode. Nevertheless, IFS 0 and HFpEF disease signature upregulation could be recovered as well, suggesting that the described cellular pathways are partially coincidental events in the murine model over time.

### Macrophage activation in single-cell transcriptomics and flow cytometry

The cellular and molecular pathways that lead to the activation of fibroblasts in HFpEF are unknown. One possible role could be attributed to macrophages which have been discussed as a crucial modulator of fibroblast activity in HFpEF [53, 70]. Our single-cell data suggested evidence for macrophage involvement (Fig. 1F), therefore we further investigated macrophage phenotypes in the HFpEF model.

**Fig. 5:**
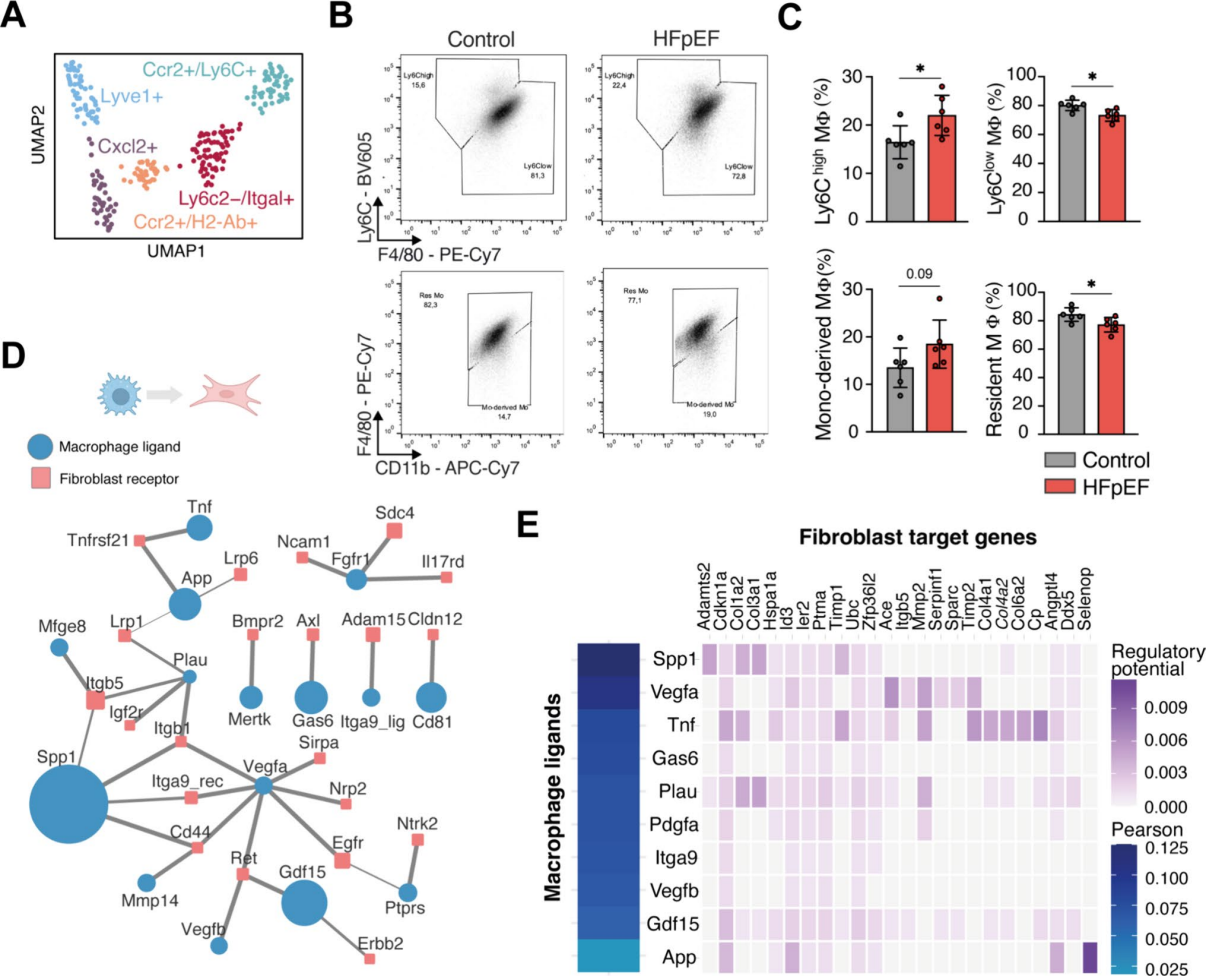
Macrophage engagement in HFpEF. **A** UMAP embedding of integrated and clustered macrophages from control and HFpEF mice. **B** Representative flow cytometry plots of Ly6C^high/low^ monocytes and macrophages (MΦ) (top row), monocyte-derived/resident MΦ (bottom row) in HFpEF vs control mice. Cells were gated on CD45^+^Lin^+^CD11b^+^ cardiac cells. **C** Quantification of flow cytometry results. Statistical analysis using *t* test, bar graphs indicate mean ± SD, *n* = 6/group *p < 0.05, *ns* not significant. **D** Ligand-Receptor network based on LIANA, receptors in fibroblasts shown in red, blue depicts ligands from macrophages. Node size visualizes effect size of upregulation in HFpEF mice, edge width visualizes HFpEF specificity (see methods). **E** Pearson correlation of top predicted ligands in HFpEF (from **D**) in NicheNet (left panel). Top NicheNet ligands and their regulatory potential with fibroblast target genes (right panel)

We identified four cardiac macrophage and one Ccr2^+^ /Ly6c^+^ monocyte cluster (Fig. 5A), the latter expressed high levels of marker genes of inflammatory monocytes (Ly6c1 and Ccr2) and fibrosis mediating genes (Fn1, Thbs1 and Vim). The macrophage cluster differed in marker expression of monocyte-derived or resident macrophage-related genes (Supp. Fig. 9A). To estimate reliable macrophage compositions without a relevant sampling-error, which might limit interpretation of our single-cell data due to low cell counts, we performed flow cytometry experiments of the entire ventricular tissue. The flow cytometry data reveal a significantly expanding proportion of pro-inflammatory Ly6C^high^ monocytes/macrophages next to decreased Ly6C^low^ macrophages (Fig. 5B, C). Gating macrophages according to their F4/80 and CD11b expression showed a significantly reduced proportion of resident (not monocyte-derived) macrophages, potentially driven by expanded monocyte-derived macrophages, which did not reach statistical significance (Fig. 5B, C, Supp. Fig. 9B). Total counts of tissue leukocytes, granulocytes and macrophages subsets did not differ significantly (Supp. Fig. 9C–F). To analyze whether the shift to pro-inflammatory macrophages is related to a splenic activation as a major source for myeloid cells in acute tissue injury [54, 55], we performed flow cytometry analysis of spleen and also peritoneal macrophages as potentially contributing inflammatory compartments following HFpEF diet. Neither splenic nor peritoneal macrophages showed a significant induction of pro-inflammatory subsets, such as Ly6C^high^ spleen or small peritoneal macrophages [16] (Supp. Fig. 9G-J). Taken together, next to expanding Cxcl2^+^ , Ccr2 + /H2-Ab^+^ and Lyve1^+^ macrophages in single-cell transcriptomics (Supp. Fig. 9 K), we observed local changes in HFpEF cardiac tissue towards a pro-inflammatory monocyte/macrophage composition, but not systemically in splenic and peritoneal compartments.

### Cell–cell communication between macrophages and fibroblasts

The observed fibroblast activation and inflammatory response of macrophages led us to hypothesize about potential cellular communication between both cell types. We used LIANA [25] to score ligand-receptor (LR) interactions between macrophages and fibroblasts in control and HFpEF mice (see methods, Supp. Fig. 10A, B). Top predicted LR pairs were upregulated in HFpEF (Supp. Fig. 10C) and included Spp1 binding CD44 or Itgb1, and Tnf binding Tnfrsf21 (Fig. 5D). To identify possible links to the HFpEF fibroblast disease signature we used NicheNet [14] to assess the regulatory potential of predicted ligands (Fig. 5E, left panel). Spp1 is predicted with regulatory potential affecting core fibrotic genes such as Col1a2, Col3a1, Adamts2 and Timp1, while Tnf ligand could be associated with basement membrane component Col4a1 and Angptl4 regulation (Fig. 5E, right panel). Expression patterns of ligands (Supp. Fig. 10D) suggest that Cxcl2^+^ macrophages could communicate via Spp1, Vegfa, Gdf15, and Plau ligands while Lyve1^+^ macrophages might secrete Gas6 and Pdgfa ligands. Tnf and Vegfb ligands were expressed in both states. We assessed regulatory patterns of these predicted LR pairs in the other HF models and found that Spp1 was induced in HFpEF and early MI and thus could constitute a mediator of the inflammatory response in both models (Supp. Fig. 10E, F).

### Protective effects of recombinant ANGPTL4

**Fig. 6:**
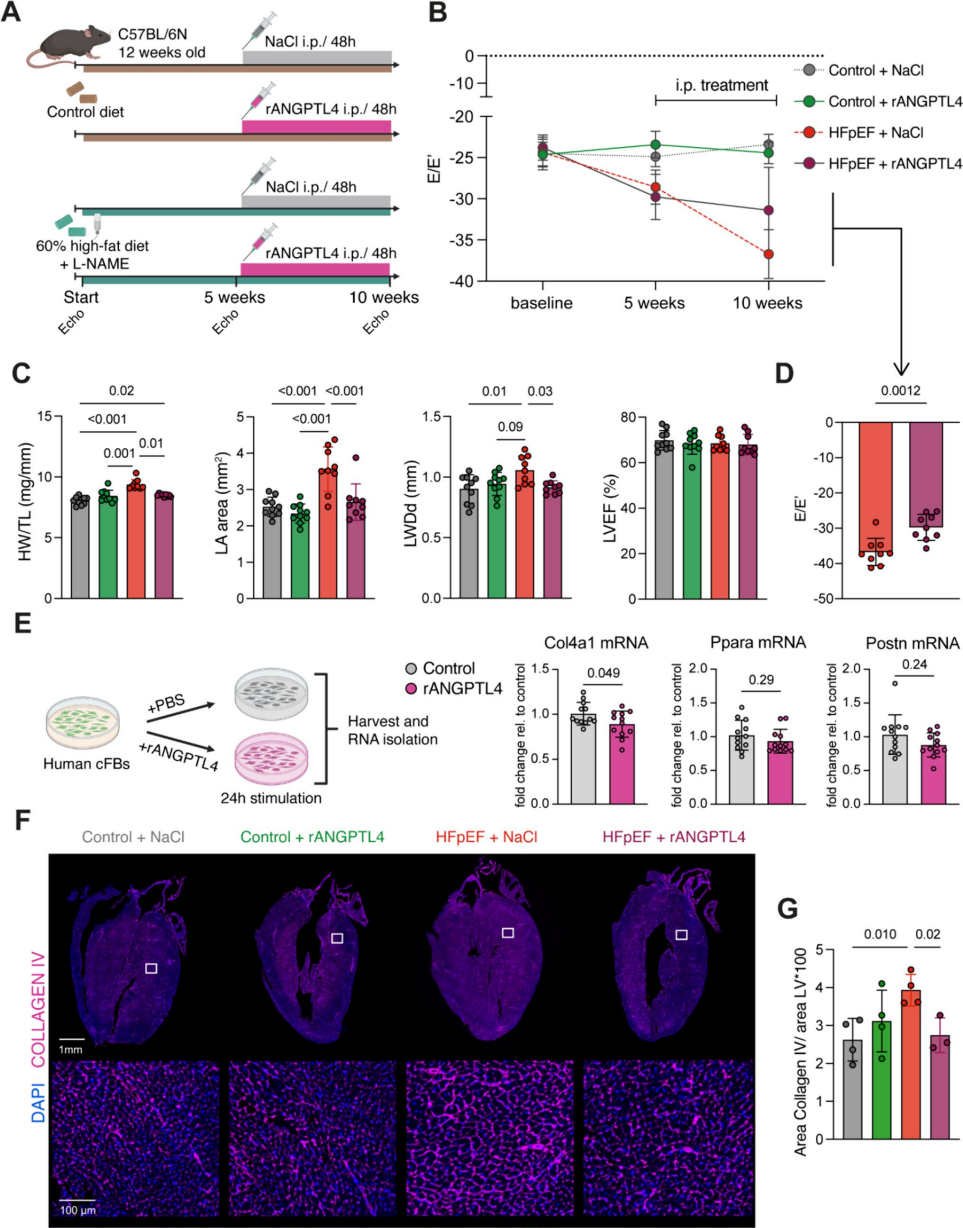
Angptl4 improves diastolic dysfunction by reducing collagen IV deposition in vivo and in vitro. **A** In vivo study design comparing recombinant murine Angptl4 peptide (rANGPTL4, 200 ng in 50 µl NaCl) vs. NaCl control (0.9%, 50 µl) administration every second day i.p. for 5 weeks starting after 5 weeks of dietary induction. Murine HFpEF induction by 0.5 g/L L-NAME and 60% high-fat diet for 10 weeks in total. Echocardiography (echo) captured cardiac systolic and diastolic function at baseline, after 5 (prior to i.p. injection start) and 10 weeks. Created with BioRender. **B** Time course of diastolic function determined by E/E’ (PW Doppler velocity across the mitral valve (E) and peak tissue Doppler at the mitral valve annulus (E’) during early diastole). *n* = 10/10/9/9. **C** Comparison of the experimental groups after 10 weeks. *n* = 10/10/9/9. One-way ANOVA with Tukey correction for multiple comparison or Kruskal–Wallis test according to normality determined by Shapiro–Wilk test. *p* values < 0.09 shown above bars, *p* < 0.05 defined as statistically significant. *HW/TL* heart weight/ tibia length, *LA* left atrium, *LVEF* left ventricular ejection fraction, *LWDd* left ventricular lateral wall diameter in end-diastole from short axis views **D** Comparison of E/E’ after 10 weeks in HFpEF mice either treated with control or rANGPTL4. *n* = 9/9, unpaired *t* test, *p* value shown above bar. **E** Experimental design (left panel) of stimulating human ventricular cardiac fibroblasts (cFBs) in vitro with 2 µg/ml human recombinant ANGPTL4 or PBS for 24 h. Resulting mRNA levels (right panel) were determined by qPCR and values indicate fold change relative to control mean. *n* = 12/12, unpaired *t* test or Mann–Whitney test according to normality determined by Shapiro–Wilk test, *p* values shown above bars. Created with BioRender. **F** Representative immunofluorescence stainings of collagen IV 1:200 (pink) and DAPI (blue). Whole heart long-axis cryo-sections depicted in the top row with white boxes indicating magnifications shown in bottom row and respective scale bars. **G** Quantification of **F** by scanning whole heart sections using a slide scanner and semi-automated analysis of the whole left ventricular (LV) tissue by normalizing the collagen IV positive LV area to total LV area using QuPath. One-way ANOVA, *p* values < 0.05 shown above bars

We described the expression pattern of Angptl4 as a state-independent marker of fibroblast activation in murine HFpEF with little expression in other interstitial cell types (Supp. Fig. 11A, B) and as a unique characteristic of HFpEF and early MI signature (Supp. Fig. 11C). We further confirmed an upregulation on protein level (Fig. 3H) and reported Ppar-ɑ and Ppar-ɣ to be active TFs as possible upstream regulators of Angptl4, together with a regulatory potential via macrophage-based TNFɑ. Angptl4 is functionally linked to inflammation, metabolism and fibrosis [9]. Thus, we hypothesized that ANGPTL4 might be involved in HFpEF pathophysiology. Aiming to study its mechanistic role in murine HFpEF, mice were treated with recombinant murine ANGPTL4 (rANGPTL4) peptide or NaCl as control i.p. every second day (Fig. 6A). This treatment was started after 5 weeks of murine HFpEF induction or respective control diet when the diastolic dysfunction began to manifest in the HFpEF group (Fig. 6B). As a result, treatment with rANGPTL4 for 5 weeks was able to rescue the phenotype as well as functional characteristics of murine HFpEF by reducing the heart weight, left atrial dilation and hypertrophy of the left ventricular wall, while no effects were observed in control diet animals treated with rANGPTL4, including an unchanged LVEF (Fig. 6C). Most importantly, rANGPTL4 was able to improve the diastolic dysfunction induced in the HFpEF model as these animals exhibited an ameliorated E/E’ ratio in echocardiography assessments following rANGPTL4 administration compared to NaCl (Fig. 6D) and no further disease progression until week 10 was observed (Fig. 6B). Based on our previous findings regarding the central role of fibroblasts and their disease-associated expression of Angptl4, we further elucidated underlying mechanisms of Angptl4 in vitro using human ventricular fibroblasts (Fig. 6E). Following stimulation with recombinant human ANGPTL4 the expression of several genes derived from our scSeq analysis was studied, including makers of fibroblast activation, such as Postn, regulators of Angptl4 (Ppar-ɑ) and different collagens (Col4a1, Col1a1). Surprisingly, only Col4 was differentially regulated upon rANGPTL4 stimulation in fibroblasts, inducing a significant downregulation (Fig. 6E). However, in HFpEF mice we observed an increasing accumulation of collagen IV next to an upregulation of Angptl4 expression. Next, we investigated the protein levels of collagen IV in hearts from mice treated with murine rANGPTL4 (Fig. 6A). In line with our initial observations, collagen IV deposition increased after 10 weeks of HFpEF diet, but treatment with rANGPTL4 was able to revert the accumulation in murine HFpEF back to healthy control levels (Fig. 6F–G). As perivascular fibrosis and basement membrane remodeling via collagen IV might impact ventricular biomechanics, these changes might be a link between improved diastolic cardiac function in rANGPTL4 treated HFpEF animals and highlight the beneficial effects of Angptl4 induction in murine HFpEF. However, further research regarding collagen IV and the exact mechanisms linking its accumulation to Angptl4 is needed. In addition, no direct or systemic metabolic effects were studied in this model, as we focused on Angptl4 regulating fibroblast function.

### Circulating ANGPTL4 levels in HFpEF vs. non-HFpEF patients

Identifying traceable markers of fibroblast activation in humans could help to assess and possibly target HFpEF remodeling at an early stage, therefore we evaluated whether ANGPTL4 could be related to HFpEF disease characteristics in human plasma.

**Fig. 7:**
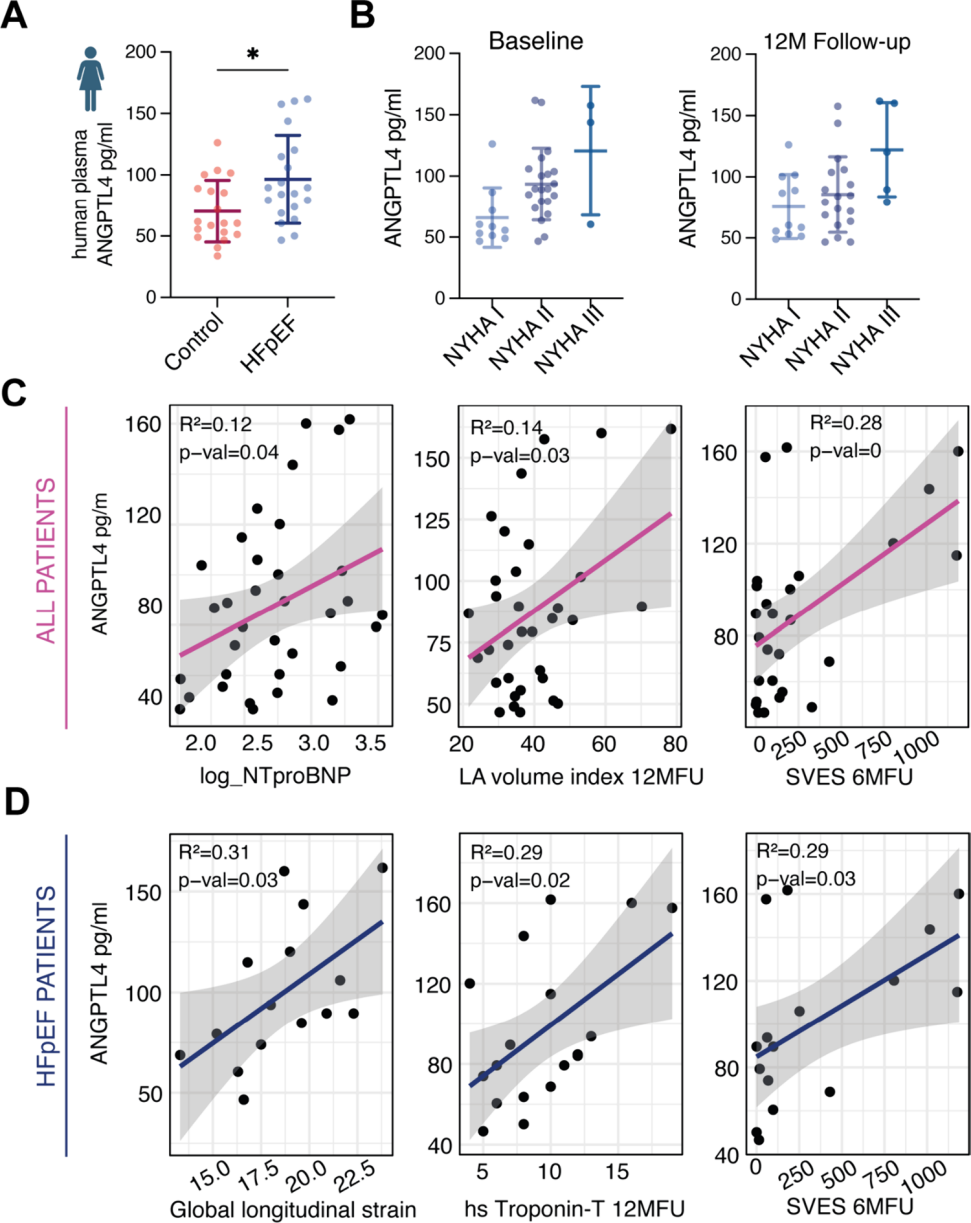
Plasma ANGPTL4 is increased in HFpEF patients. **A** Circulating levels of ANGPTL4 in human plasma samples of HFpEF and age-matched controls measured by sandwich ELISA. *n* = 19/20, Mann–Whitney *U* test, *p < 0.05. B) ANGPTL4 plasma levels in relation to NYHA functional class of all recruited patients. ANOVA, *p* value < 0.05, *n* = 10/21/3 in baseline and *n* = 11/18/5 in 12 months (12 M) follow-up. **C** Correlation of clinical parameters to ANGPTL4 circulating levels in all patients (control and HFpEF) and **D** as subanalysis only in HFpEF patients using simple linear regression. *p-val* indicates uncorrected *p* value. *hs* high sensitivity, *LA* left atrial, *MFU* months follow-up, *SVES* supraventricular extrasystoles. Plots in (**A**, **B**) display mean ± SD

We analyzed circulating levels of ANGPTL4 in 20 plasma samples of HFpEF and 20 propensity score-matched non-HFpEF (control) patients. All patients were diagnosed for symptomatic atrial fibrillation and screened for HFpEF by echocardiography, stress echocardiography, NT-proBNP, and HFA-PEFF-score [111]. Plasma samples were analyzed by ELISA, which revealed significantly higher circulating ANGPTL4 levels in HFpEF (Fig. 7A), suggesting a possible role in cardiometabolic disease. We further quantified potential associations with various clinical features of this cohort to provide hypotheses about possible functional characteristics. ANGPTL4 levels increased significantly in higher NYHA stages in all patients (Fig. 7B) and correlated significantly with NT-proBNP, but not with high-sensitivity troponin T (Supp. Fig. 11D). In a subanalysis of the HFpEF cohort, high ANGPTL4 levels related positively to counts of supraventricular extrasystoles in holter ECGs and left atrial volume index (biplane, ml/m^2^), at 6- and 12-months follow-up, respectively (Fig. 7C, D), but not at baseline indicating a potential association with disease progression as it is known that left atrial dilatation and supraventricular arrhythmias are associated with HFpEF severity [109]. Exclusively in HFpEF, but not in control patients, plasma ANGPTL4 could be associated with troponin T levels. Although ANGPTL4 seems to be related to some features of disease severity, high levels were possibly associated with a preserved global-longitudinal strain (Fig. 7D), which represents the most sensitive marker for cardiac dysfunction. These data suggested that ANGPTL4 could be part of a compensatory mechanism in HFpEF that protects from metabolic stress.

## Discussion

In this study, we provided a first comprehensive characterization of interstitial cardiac remodeling in a two-hit HFpEF mouse model on single-cell level. Deterioration of cardiac diastolic function was accompanied by increased perivascular fibrosis in murine HFpEF hearts. This phenotype was associated with a pro-fibrotic gene program in fibroblasts. By integrating single-cell atlases of two additional murine HFrEF models, we identified conserved fibroblast states across models and derived common and unique functional characteristics of fibroblast activation in HFpEF compared to HFrEF. We corroborated disease signatures in human transcriptome data, and suggested possible involvement of macrophages in the activation of fibroblasts in HFpEF. Finally, we found that Angptl4 was mechanistically involved in mediating between metabolic stress and fibrosis.

Cardiac fibrosis is a hallmark of ventricular remodeling in heart failure (HF). However, disease-specific molecular patterns of the associated fibroblast functionality in different types of HF are unknown. Here, we compared fibroblast activation in early HFpEF (cardiometabolic two-hit model), renin–angiotensin–aldosterone system activation-induced HFrEF (AngII model), early and later ischemic HFrEF (myocardial infarction model) to identify common fibroblast phenotypes across models. Among fibroblasts, different phenotypes are assumed to represent functional and/or spatial niches [90, 94]. However, a consensus and nomenclature of cell states has not been accomplished yet, in part due to shortcomings of the concept of cell states attempting to i) categorize a continuity and ii) distinguish between a more transient functional nature of a state or a cell differentiation [90]. By integrating multiple studies, we provided a catalog of conserved cardiac fibroblast cell phenotypes in heart failure, possibly representing the hallmarks of cardiac fibroblast function, including ECM production (IFS 0, 3), secretory function (IFS 1), immune system modulation (IFS 6,2,7), migration (IFS 2) and tissue homeostasis (IFS 4, IFS 5).

Despite this functional diversity, ECM remodeling and collagen deposition was a common fibroblast task across models, reflecting fibrosis as disease characteristic in each HF model. In HFpEF fibroblasts, metabolic stress, heat-shock proteins and glycosylation of proteins were accompanied by upregulation of ECM components, in particular basement membrane compounds. The basement membrane represents a highly active ECM that underlies many cell types such as ECs and SMCs and provides a scaffold that connects cardiomyocytes to the ECM [12]. Functionally, it plays an important role in angiogenesis, mechanotransduction and cell differentiation [23]. The role of the basement membrane in HFpEF has not been sufficiently explored yet, but its modulation of laminins has been suggested to cause gene expression changes in cardiomyocytes related to increased stiffening [45]. Interestingly, HFpEF shared proinflammatory features like hypoxia and TNFα pathway activity with early MI fibroblasts, while late MI and AngII fibroblast displayed highest TGFꞵ activity. In addition, we observed an expansion of pro-inflammatory Ly6C^high^ monocytes/macrophages in HFpEF hearts and predicted that a mutual activation occurs in the cross-talk with fibroblast via Spp1 and TNFα. Ly6C^high^ macrophages have been associated with diastolic dysfunction before [48, 52], while SPP1 has been reported as a marker for macrophage activation after acute cardiac ischemia [46, 73]. TNFα has been closely studied in heart failure before, and was suggested to be one of the mediators of systemic inflammation in HFpEF [38, 73]. Here we present additional evidence for the involvement of these ligands in murine HFpEF, that could associate with the described interstitial fibrosis and deserve further investigation.

Single-cell transcriptomics enable a deeper characterization of cell type function in disease by regarding the division of labor between cells and their functional or spatial niches [4, 5, 105]. We demonstrated that fibroblast activation is a mixture of compositional and transcriptional shifts in all HF models with the strongest transcriptional shift following MI, possibly suggesting that acute tissue injury induces stronger population wide cell responses. Besides these transcriptional shifts, prioritized states were identified in each model, suggested by high transcriptional shifts co-occurring with compositional shifts in these states. We found that during early MI, migratory myofibroblasts (IFS 2), matrifibrocytes (IFS 3) together with other proinflammatory states (IFS 6 and 7) were prioritized, in contrast to AngII and late MI which showed convergence of their profibrotic disease signatures, suggesting that independent of the initial type of tissue damage a conserved fibroblast response is evoked over time in line with reported findings on convergence of transcriptomic profiles of end-stage ischemic and dilated cardiomyopathy [75, 88]. The early HFpEF-associated fibrosis might differ from these respective HFrEF-like remodeling processes, as we found little disease related signal in matrifibrocytes, but identified homeostatic fibroblasts (IFS 0) and basement membrane remodeling as key characteristics. Collagen I deposition is crucial for pro-fibrotic ECM remodeling and has been described as a characteristic of matrifibrocyte activity. In early HFpEF, the division of labor of collagen I expression was shifted from a state-dependent task to a general fibroblast task. This could likely be associated with the extent and composition of the observed cardiac fibrosis (considering the more abundant ECM after MI or AngII treatment compared to the HFpEF model). However, a subsidiary role of matrifibrocyte activation in metabolically driven fibrosis has been suggested before [108] [39] [7]. Our data further supported this by demonstrating that no relevant FAP expression was observed in HFpEF hearts in contrast to the previously described upregulation in acute MI and AngII/PE [6, 97]. Thus, we propose that metabolically driven fibrosis could be characterized by a particular fibroblast activation pattern that results in moderate interstitial fibrosis, possibly with different composition and function.

The contextualization of fibroblast activation with other HF models also allowed us to focus on fibroblast markers like Angptl4 that could not be found in the HFrEF signatures of late MI and AngII. Angptl4 constitutes a functional link between metabolic, inflammatory, and fibrotic mechanisms which are all closely related to HFpEF pathophysiology. It acts as a secreted matricellular protein known to regulate fibroblast activation and immune mediators next to controlling fat metabolism by inhibiting lipoprotein lipase [9, 19, 95]. Due to its broad biologic involvement and distinct roles of the N- and C-terminal fragments (at least in humans), the function of Angptl4 in different disease contexts is ambiguous. Pro-fibrotic effects of Angptl4 have been reported in lung [82] and kidney fibrosis [91], while in liver fibrosis Angptl4 acted in a protective manner [96, 109]. In atherosclerosis, loss of function mutation of ANGPTL4 in humans was associated with an increased risk [24]. However, a global Angptl4 knockout in mice found protective effects [3], while a hematopoietic cell-specific knockout showed detrimental effects on atherosclerosis [10]. In the heart, Angptl4 has been shown to enhance reparative functions of cardiac macrophages following injury [21], together with antifibrotic effects especially in the atria [110]. In the context of high-fat diet or oral lipid load, Angptl4 knockout mice showed enhanced oxidative stress, therefore the upregulation of Angptl4 was interpreted as a protective mechanism against lipid overload [37]. Importantly, in cardiomyocyte-specific Angptl4 overexpression, limited fatty acid availability caused spontaneous ventricular dysfunction in mice [107]. Here, we reported that fibroblasts upregulated Angptl4 in murine HFpEF with an accompanied estimated activation of transcription factors Ppar-ɑ, Ppar-ɣ, Hif1ɑ and hypoxia and TNFα pathways. We further demonstrated that diastolic dysfunction and cardiac hypertrophy could be prevented in HFpEF mice by administration of recombinant ANGPTL4, which was also associated with reduced collagen IV deposition. Thus, in line with previous cardiac studies, we suggest that upregulation of Angptl4 ameliorated the detrimental effects of oxidative stress and HFD. Next to expected systemic metabolic regulatory functions, our in vitro studies revealed that human fibroblasts, stimulated with recombinant ANGPTL4, downregulated Col4a1, suggesting additional direct effects on fibroblast activation and thus interstitial and perivascular fibrosis. Taken together, we contributed to elucidate Angptl4 as a possible key regulator in cardiometabolic HFpEF. However, the in-depth molecular cascade and distinctions between systemic metabolic and direct local effects on fibroblasts and immune cells in HFpEF deserve further investigation.

Regarding a potential translation of these results to human disease, we demonstrated that the fibroblast disease signature could be recovered in public transcriptomic data of HFpEF patients, who underwent coronary artery bypass graft surgery. Although this patient collective does not represent a typical HFpEF cohort, other myocardial gene expression studies were not publicly available. Nevertheless, our analysis suggested that at least some characteristics of the metabolically induced fibroblast phenotype is conserved across species. More importantly, we also found that ANGPTL4 plasma levels were elevated in HFpEF patients and related to the burden of supraventricular extrasystoles and cardiac biomarkers, but presented an interesting association of higher ANGPTL4 levels with a preserved global-longitudinal strain, a sensitive marker of cardiac function. The latter might imply potential protective effects as seen in mice, as the global-longitudinal strain predicts outcome in HFpEF patients [13]. Elevated ANGPTL4 levels have been reported in the plasma of both HFpEF and HFrEF patients [57], suggesting that more phenotypically fine-grained plasma studies are needed to dissect the association of ANGPTL4 with the HF phenotypes and relevant comorbidities like obesity and atherosclerosis.

The main limitations of our study relate to the sample size and cell number of the single-cell experiment. Subtle disease changes, such as gene programs occurring in more rare cell types or cell states, were probably not detectable. At the same time, our statistical approach for differential expression and composition analysis might result in a higher rate of false positives than more robust approaches that rely on higher sample size. However, multiple confirmation experiments suggested that disease signatures were reproducible in other data. Our study design focused on early changes of the murine cardiometabolic HFpEF model. As a longer dietary regimen might lead to further disease progression, we cannot provide insights into potential dynamics of the reported cellular disease signatures on single-cell level. A potential role of matrifibrocytes during later stages of the HFpEF model was suggested by upregulation of matrifibrocyte markers in bulk transcriptomics. However, it is unclear which time point of the murine model resembles most closely to human HFpEF. In addition, the HFD/L-NAME model does not mimic all human HFpEF characteristics as female sex is protective of diastolic dysfunction [98] and thus only male mice were used. Corroboration in human bulk transcriptome demonstrated that matrifibrocyte markers are upregulated in human HFrEF, but not in HFpEF patients. Additional validation of these findings in larger human HFpEF studies could not be accomplished, due to the small number of publicly available datasets of gene and protein expression in human HFpEF.

Pharmacomodulation of detrimental fibrosis has been mainly unsuccessful in the past. As the biologic understanding and dissection of disease-specific fibroblast activation improves, better targeted anti-fibrotic therapies might come within reach [41]. To summarize, we provided a first description of adverse interstitial remodeling in HFpEF at a single-cell level. Our work generated new insights into distinct and common features of cardiac fibrosis in murine heart failure including the protective potential of Angptl4 and might serve as a valuable resource for the scientific community to identify disease-specific treatment strategies for HFpEF in the future.

## Online methods

### Animals

All animal experiments were conducted in agreement with the animal welfare guidelines and German national laws. All animal procedures and study protocols were authorized and approved by the responsible authority (permit No. G-252/20, G-121/21 and G-282/21, Regierungspräsidium Karlsruhe, Baden-Württemberg, Germany). C57BL/6N male mice, obtained from Janvier Labs, were used at an age of 10 weeks. Mice were kept at 23 °C ambient temperature and in 12 h light/dark cycle ﻿and had unrestricted access to food (D12450B, control diet rodents 5% fat and D12492, rodent 60% high-fat diet for the HFpEF group, Ssniff) and water. HFpEF was induced as reported previously [84]. Briefly, Nω-nitro-l-arginine methyl ester (L-NAME, 0.5 g/l, Sigma–Aldrich), adjusted to pH 7.4, was supplied by the drinking water in light-protected bottles for the indicated time. Figure 1A (left panel) and Supp. Fig. 1B + D included n = 11 controls (7, 10 and 15 week control diet), n = 8 7-week HFpEF, n = 9 10-week and n = 7 15-week HFpEF animals. For murine recombinant ANGPTL4 (R&D, 4880-AN-050) peptide treatment, HFpEF was induced as described above and after 5 weeks i.p. injections of either 200 ng rANGPTL4 (in 50 µl NaCl) or NaCl (50 µl) as control. Injections were continued for another 5 weeks every second day. The experiment was started with 10 mice/ group; however, 2 mice had been removed from the experiment due to a skin wound (*n* = 1) or inadequate body weight gain (*n* = 1) resulting in *n* = 10/10/9/9 animals per group.

Acute MI paraffin embedded sections were derived from a C57BL/6N mouse 28 days after minimal-invasive occlusion of the LAD, as described previously [87]. Infarct size of large MI can be gathered from Supp. Fig. 8A.

### ﻿Echocardiographic measurements

Transthoracic echocardiography was performed on a VisualSonics Vevo 2100 system equipped with MS400 transducer (Visual Sonics). Left ventricular (LV) parasternal long-axis and short-axis views at the mid-papillary muscle level were acquired by induction (4 vol%) and short maintenance (0.5–1.5 vol%) of isoflurane anesthesia. LV end-diastolic volume (LVEDV), fractional area change (FAC), LV fractional shortening (FS) and LV ejection fraction (LVEF) were obtained at a heart rate between 500 and 600 bpm. Parasternal long-axis traces were used to calculate the global-longitudinal strain with a software- and speckle-tracking algorithm (VevoStrain software,Visual Sonics). Borders of the endocardium and epicardium were subsequently traced before a semi-automated strain analysis was performed by the software. To measure left atrium (LA) diameter and left ventricular lateral wall diameter (LWDd) in awake mice to avoid isoflurane-induced cardiac depression as confounder, a modified parasternal LA-view was captured and LA size circled using respective tools from the Vevo Lab software. LWDd was measured in short axis views in end-diastole as a marker for cardiac hypertrophy.

For diastolic function, mice were anesthetized under body temperature-controlled conditions and maintenance (1.5–3 vol%) of isoflurane anesthesia aiming to keep the heart rate in the range of 400–450 bpm. Apical four-chamber views were obtained and pulsed-wave and tissue Doppler imaging at the level of the mitral valve performed to record the following parameters: peak Doppler blood inflow velocity across the mitral valve during early (E) and late diastole (A), isovolumic relaxation time (IVRT) and peak tissue Doppler of myocardial relaxation velocity at the mitral valve annulus during early diastole (E’). Analysis was performed with VisualSonics Vevo Lab software, using semi-automated LV tracing measurements for LVEF and FS. All parameters were measured in at least three cycles, and means were presented. GLS measurements could not be performed retrospectively in the animals used for scRNASeq due to bad semi-automated tracing of the images.

### Single-cell RNA sequencing

Sample preparation and sequencing of murine cardiac interstitial cells was performed according to the detailed protocol published previously with only minor modifications[30]. In brief, rapidly after sacrificing the animals by cervical dislocation, the still beating heart was directly placed in ice-cold HBSS where atria and large vessels were dissected. After chopping the heart into small pieces, enzymatic digestion was initiated in two rounds of 15 min duration at 37 °C using collagenase type II (﻿Worthington Biochemical Corporation, ﻿# LS004177). The single-cell suspension was subsequently passed through a 40 µm cell strainer, washed and red blood cell lysis performed. Dead cells were removed by Dead Cell Removal MicroBeads ﻿ (Miltenyi Biotec, 130–090-101) binding to MACS ﻿LS columns (Miltenyi Biotec, 130–042-401). Prior to loading the 10 × platform, live nucleated cells (DRAQ5^+^, propidium iodine^−^) were sorted using a FACSAria™ IIu (BD) cell sorter. Following washing and resuspending in PBS, cells were counted manually using trypan blue and a Neubauer chamber. We aimed to load about 5,000 cells per lane on a Chromium Next GEM Chip (10 × Genomics, 1000127), that was placed into a 10X Chromium Controller (10X Genomics). The cDNA output was amplified and library construction was performed according to the manufacturer’s instructions using the Chromium Next GEM Single Cell 3' Kit v3.1 (10 × Genomics, 1000269) and Dual Index Kit TT Set A (10 × Genomics, 1000215). Respective library quantification and quality controls were performed using an Agilent 2100 Bioanalyzer and in addition a Qubit HS Assay. Indexed libraries were equimolarly pooled resulting in two sequencing runs (control1 + HFpEF1; control2 + HFpEF2) using a High Output kit v2.5 (Illumina, 20024907) and a NextSeq® 550 (Illumina) sequencer.

### Data preprocessing and QC

The resulting single-cell RNA-seq outputs were processed using CellRanger provided by 10 × genomics. Count data were processed sample wise with the following filters: > 300 Feature numbers, < 25% mitochondrial genes, < 1% ribosomal genes and > 500 RNA counts. Doublet scores were calculated with the R-package *scDblFinder *[61] and only predicted singlets were kept. We further calculated a dissociation score by estimating expression of dissociation associated gene expression [68] with Seurat’s [40] AddModuleScore function and we removed cells above the 99% quantile. Data was log-normalized. Samples were clustered individually by selecting the 3,000 highest variable genes with the FindVariableFeatures function from the *Seurat* package. From the overlap of these lists, the top 3,000 genes were selected to calculate principal components (PCs). Top 30 PC embeddings were adjusted with *harmony* R-package, with samples as covariates. In the resulting integrated feature space the nearest neighbor approach and graph-based Louvain algorithm implemented in *Seurat* was used to cluster cells and stepwise test optimal cluster resolution (from 0.1 to 1.6 in 0.1 steps) and computing silhouette widths. Celltype markers were calculated with the FindMarkers function with default parameters (Wilcoxon test) in *Seurat* and cell types were manually annotated based on known canonical markers.

We removed four distinct small clusters that were inconclusive for different reasons, i.e. high expression of mitochondrial genes, expression of multiple cell type markers, consistently low RNA and Feature counts. After removal, the integration process was repeated and a final atlas was created.

### Composition analysis

We tested if cell type or state composition changes between groups are meaningful by implementing a permutation approach to estimate a null distribution. For each individual cell, we considered the sample it came from and the cell type label it was assigned. From this table, we created 1000 permutations. For each permutation run we calculated the cell proportions for each sample and calculated the mean proportion per cell per group (control, HF), from which the difference in cell proportion was calculated as test statistic. By calculating the proportion per sample and not per group, we simulated unequal cell numbers in samples. The resulting 1000 random cell proportion differences are an estimate for a null distribution (Supp. Fig. 3A). All distributions passed Shapiro–Wilk test for normality (*p* > 0.05). We calculated the area under the normal curve from the mean and standard deviation of the null distribution to estimate the probability of observing the actual measured proportional difference (Supp. Fig. 3B).

### Sample distance

To prioritize cell types displaying disease signatures we calculated distances between cell types per sample [71]. First, highly variable features were calculated per cell type with *FindVarFeature* function from *Seurat* and the top 1000 features were selected for distance calculation. For each cell type and sample, pseudobulk profiles were TMM normalized and voom transformed with the *edgeR* and *voom* R-package and cosine distances were calculated. We calculated median sample distances within groups and between groups to assess the distance ratio. Cell types with distance ratio below 1 show higher sample distances between groups than within groups and are candidates for differential gene expression analysis.

In addition to sample distance, we applied the R-package *Augur *[89] to train random forests to classify the experimental group (control vs. heart failure) of individual cells. We used the *calculate_auc* function with default parameters.

### Differential gene expression analysis

To control for different absolute numbers of cells per sample we subsampled the total number of cells to the lowest cell number in a sample. For these cells, we calculated differentially expressed genes with *FindMarker* function from *Seurat* R-package. To ameliorate sampling effects we repeated this subsampling process 5 times, and reported the gene intersection of genes with Benjamini–Hochberg-corrected *p* value < 0.05 and absolute log2FC > 0.1. Upregulated genes were considered as the disease signature of the respective HF model.

### Cell state analysis

To identify cell states in macrophages, we subset each sample to macrophages and reintegrated samples by following the same steps as described above. For fibroblasts, the integrated atlas was used to calculate meaningful distances between cells via construction a nearest neighbor graph. Cell states were then defined by the Louvain clustering algorithm implemented in *Seurat’s FindCluster* function, and optimal cluster resolution (0.1 to 1 in 0.1 steps) was determined by selecting the resolution with maximal silhouette width. Cellstate interpretation was aided by processing cell states reported in [15] from steady and perturbed state cell markers.

### Functional analysis

We performed functional analysis of top 100 cell state markers and fibrotic signatures. Overrepresentation analysis was performed with *enrichR,* with GO-molecular function and biological function terms. Additionally, functional gene sets were acquired from MSIG DB and subjected to hypergeometric testing. Pathway analysis was performed with PROGENy [86]. To calculate cell state pathway activities, we summed up cells per cell state to create pseudobulk profiles, which were analyzed for pathway activities. For the integrated atlas we relied on running pathway analysis per cell to not sum uncorrected counts to pseudobulks. For study comparison we used log fold change as an effect size reported per study to calculate progeny scores. We used TF regulons obtained from *DoRoThEA *[11, 36] and the *decoupleR *[11, 36] R-package to estimate TF activities. We used univariate linear models to estimate TF activity on logFC vectors from different studies. Finally, we calculated module scores which are weighted expression means for genesets with the *AddModuleScore* function in *Seurat.*

### Study integration

Two additional 10 × Genomics scRNAseq datasets were analyzed by downloading raw FASTQ files and processing via cell ranger pipeline as described above. Sample integration was performed via canonical correlation analysis as implemented in *Seurat*. Unsupervised clustering and cluster marker assessment was used to identify fibroblasts in each study, which were subset to perform study integration. We integrated fibroblast cell data from three datasets via calculating highly variable features in each dataset, using 3000 overlapping features of all datasets. We used *Harmony* with study and sample ID as covariates for dataset integration. Downstream analysis was performed as described above. Integrated data was reclustered to identify cell states and markers for each cluster were calculated based on log transformed data. To evaluate integration performance we ensured that each study contributed cells to each cluster (Supp. Fig. 6). To quantify batch effects from different studies, samples and experimental groups, we calculated a batch mixing score based on average silhouette width as proposed previously [60]. A score of 1 represents a balanced integration while 0 represents strong batch effect conservation. The Integrated fibroblasts atlas yielded a batch score of ~ 0.99 for study labels, ~ 1 for group labels and ~ 0.97 for sample labels. To avoid study batch effects in differential expression analysis, we calculated differentially expressed genes between control and disease models per study. We performed downsampling to equalize cell numbers per sample as described earlier. We collected genes that appeared in at least 4 of 5 downsampling runs by passing Benjamini–Hochberg adjusted Wilcoxon *p* value < 0.05.

### Assessing state dependency of transcriptional shifts

To estimate transcriptional shifts of disease signatures, we estimated how well these signatures separate healthy and disease cells within an assigned cell state. We first calculated gene set scores for each cell of the respective HF model via the *AddModuleScore* function from *Seurat* R-package (Supp. Fig. 6A). The difference of these gene set scores was then assessed by calculating the area under the receiver operator curve (AUROC) (Supp. Fig. 6B) as a metric for the transcriptional shift within a state.

For a single gene being expressed in a state-dependent manner we expected that expression levels would vary between states. To quantify this dependency, we fit ANOVA models for each gene of the disease signatures by modeling their expression value by IFS category (gene X ~ IFS) and extracted the explained variance of the model (eta^2^ values). To compare these with disease related variance we fit ANOVA models for the same genes but with group labels (gene X ~ group). The ANOVAs were calculated for all HF models separately.

### Cell–cell communication

We used two approaches to estimate cell–cell communication. First, we performed ligand-receptor (LR) analysis with the method aggregation tool *LIANA *[25]. Second, we used *NichNet *[14] to connect ligands with receiver cell gene expression [14]. To lower the false positive rate of LR pairs, we only analyzed macrophages and fibroblasts as both cell types displayed the strongest disease response. We ran *LIANA* on control and HFpEF mice separately. Within each group we aggregated ranked LR pairs from 6 LR tools with the *aggregate_l* function in *LIANA*. We assumed that an LR pair is specific for HFpEF if it is ranked low in control and high in HFpEF mice. To quantify this we calculated a HFpEF specificity score (*S*) by: $S = (1- RankCT)* RankHF$.

We selected top 30 LR pairs based on *S* and filtered for ligands being expressed by more than 0.15 percent in HFpEF sender cells and to be upregulated > 0.1 log2FC; receptors were filtered for expression percentage > 0.15 in HFpEF receiver cells (Supp. Fig. 9E). The top ligands from macrophages were used as input for *NicheNet* to model regulatory potential for the HFpEF disease signature. Since the prior knowledge graph in NicheNet is undirected, directional regulation of target genes cannot be modeled. For this reason we used up- and downregulated genes in HFpEF fibroblasts as the target cell gene set.

### Human bulk RNA-seq

For human HFpEF bulk analysis, raw count data was downloaded from European nucleotide archive, accession number E-MTAB-7454. We filtered genes by a minimum of two RNA counts in at least 30% of samples per experimental group. We TMM normalized samples and voom transformed for variance stabilization and performed DEA with *limma* and *edgeR* R-packages. HFrEF bulk data from the *Reference of the Heart Failure Transcriptome* (ReHeaT) [75] is available at: https://zenodo.org/record/3797044#.XsQPMy2B2u5. We selected the top 500 genes for overrepresentation analysis.

### Murine bulk RNA-seq of different timepoints from HFpEF hearts

Mice of the HFpEF model were used after 10 (*n* = 4) and 15 (*n* = 4) weeks of diet protocol and compared to mice that received control diet (*n* = 3). After killing the animals by cervical dislocation, the still beating heart was directly placed in ice-cold HBSS where atria and large vessels were dissected and immediately placed into liquid nitrogen and stored at -80°. The tissue was homogenized and total RNA was isolated using TRIzol (Thermo Fischer Scientific). Quantification and quality controls were performed by DS-11 spectrophotometer (DeNovix) and 5300 Fragment Analyzer System (Agilent) and samples used with RNA Integrity Number (RIN) > 8 (mean RIN 9.2 ± 0.5 SD). Library construction and sequencing was performed by the GeneCore Facility at EMBL Heidelberg. Briefly, libraries were prepared from 1 µg total RNA using respective Illumina mRNA Kits according to the manufacturer’s instructions. Samples were multiplexed and single-end sequencing performed on an NextSeq 500 (Illumina). Raw BCL data were demultiplexed and then converted to FASTQ files. Reads were aligned via ArchS4 pipeline implemented in BioJupies [99]. We filtered lowly expressed genes and normalized samples using the trimmed mean of M-values (*edgeR* [78]) and subsequent variance-stabilizing transformation (limma voom) and performed differential expression analysis (*limma *[77]). Resulting *t* values were used for enrichment analysis via run_ulm function from *decoupleR *[11, 36] R-package.

### Flow cytometry

To obtain a single-cell suspension, the removed hearts were minced and digested in 450 U/mL collagenase I, 125 U/mL collagenase XI, 60 U/mL DNase I, and 60 U/mL hyaluronidase (MilliporeSigma) for 1 h at 37 °C under agitation. Cells were washed, counted and diluted to 100 µl per 10 × 10^6^ cells prior staining. The whole cardiac sample was further proceeded for and passed through multiparameter flow cytometry analysis. An additional control sample was used for unstained and fluorescence-minus-one gating controls. Spleens were passed through a 40 µm cell strainer and washed prior to further staining. Peritoneal macrophages were collected directly after sacrificing the animal by peritoneal lavage performed by injecting 5 ml of ice-cold PBS with 3% FCS using a 27G needle. After an abdominal massage for 30 s the fluid was collected by a plastic pipette through a small incision of the abdominal wall. Cells were kept on ice, washed and resuspended for 1:100 antibody staining. For all samples, Fc receptor blocking was performed for 10 min prior to fluorescent antibody staining using anti-CD45-PerCP-Cy5.5 (BD Biosciences, clone 30-F11), anti-CD11b-APC-Cy7 (BD Biosciences, clone M1/70), anti-Ly6C-BV605 (Biolegend, clone AL-21), anti-F4/80-PE-Cy7 (Biolegend, clone BM8), anti-MHCII-BV421 (BD Biosciences, clone M5/114.15.2), anti-CCR2-APC (R&D Systems, clone #475301). For lineage exclusion PE-conjugated anti-Ter119 (BD Biosciences, clone TER-119), anti-NKT (BD Biosciences, clone U5A2-13), anti-B220 (BD Biosciences, clone RA3-6B2), anti-CD49b (BD Biosciences, clone DX5), anti-90.2 (BD Biosciences, clone 53-2.1) and anti-Ly6G (BD Biosciences, clone 1A8) antibodies were used. Gating strategies and representative plots are presented in the supplementary figures (Supp. Fig. 11). Flow cytometry was performed on a FACSCelesta (BD) and data analysis conducted by FlowJo (BD) software.

### Histological analysis

Harvested hearts were rinsed in PBS, and fixed for 7 days in 10% buffered formalin at room temperature. Hearts were subsequently dehydrated, paraffinized, and sectioned (5 μm). Cardiac fibrosis was assessed using the cardiac muscle Picrosirius-Red Stain Kit (Abcam, ab245887) according to the manufacturer's instructions. Quantification was performed on total heart scans by AxioScan 7 (Zeiss) with high resolution as demonstrated (Supp. Fig. 1 M). Comparable areas were analyzed containing one blood vessel of similar size in left ventricular tissue. Absolute MFI values/ 1000 were presented. Immunohistochemistry (IHC) was performed using heat-mediated antigen retrieval in sodium-citrate buffer at pH 6.0 and an anti-rabbit secondary antibody containing HRP/DAB Detection IHC Kit (abcam, ab64261) according to the manufacturer's instructions. The following primary antibodies were used: anti-ANGPTL4 (#710,186, ThermoFisher Scientific, clone 1HCLC), anti-CILP (CAU24345, Biomatik), anti-FAP (ab207178, Abcam). Slides were mounted and imaged using a brightfield (SP2, Leica) and fluorescence microscope (Axio Observer, Zeiss).

### Immunofluorescence

For immunofluorescence staining, in TissueTek embedded and frozen heart-derived cryostat sections were fixed in 3,7% PFA, permeabilized for 20 min, blocked with 5% BSA for 1 h, and stained overnight with anti-CollagenIV (1:200, ab6586, Abcam) and the following day for one hour with goat anti-rabbit AlexaFluor594 (1:400, ab150080, abcam) secondary antibody. Nuclei were stained with a DAPI-containing mounting medium, covered and images captured using a Axio Observer (Zeiss) fluorescence microscope or using an automated slide-scanner (Axioscan7, Zeiss). Total heart scans were semi-automatically analyzed using QuPath software (QuPath, v0.5.0) trained to circle LV tissue only. The positive stained area was normalized to the total area of the left ventricle in long-axis cut sections.

### Human ventricular fibroblast stimulation

Human primary cardiac ventricular fibroblasts (Lonza, #CC-2904) at passage 6 were cultured according to the manufacturers recommendations and respective basal and growth media. One day after plating of 300,000 cells/well in 6-well plates on hydrogels (Softwell, 50 kPa) with lower stiffness than plastic surfaces to mimic a diseased heart, cells were treated either with recombinant human ANGPTL4 (R&D, #4487-AN-050) 2 µg/ml or PBS for 24 h in fibroblast growth medium at 37 °C. Prior to RNA isolation, the supernatant was collected and cells were washed twice in PBS.

### Quantitative real-time PCR

Total RNA was isolated from human cardiac fibroblasts using the RNeasy Mini Kit (Quiagen, 74,104) according to the manufacturer’s instructions. Following RNA extraction, the quality of RNA samples was determined by measuring the concentration, 260/280 and 260/230 ratios with a DeNovix DS-11 Series Spectrophotometer. Subsequently, RNA was reverse transcribed to cDNA using the iScript cDNA Synthesis Kit (Bio-Rad Laboratories, 1,708,891). For real-time quantitative PCR, SYBR Green (Bio-Rad Laboratories, 1,725,124) and the following primers were used: Col4a1 (forward primer: CTGCCTGGAGGAGTTTAGAAG, reverse primer: GAACATCTCGCTCCTCTCTATG), Postn (forward primer: AGATCCGTGAAGGTGGTTTG, reverse primer: GTCTTTGAGACGCTGGAAGG), PPARy (forward primer: AGCCTGCGAAAGCCTTTTGGTG, reverse primer: GGCTTCACATTCAGCAAACCTGG), GAPDH (forward primer: GGCTCATGACCACAGTCCAT, reverse primer: GCCTGCTTCACCACCTTCT). Relative fold changes of gene expression levels were normalized to GAPDH mRNA and calculated using the 2^–∆∆Ct^ method.

### FAPI PET–CT

We performed murine PET–CT imaging with 68 Ga-FAPI-46 as described previously [56]. Briefly, the radiotracer (ca. 13 MBq), produced and approved for patient application, was injected via the tail vein of isoflurane anesthetized mice and 10 min static images were recorded using a small-animal PET–CT scanner (Inveon; Siemens).

### HFpEF patient population

The study protocol is in accordance with the declaration of Helsinki and has been approved by the local ethics committee of the University Hospital Heidelberg and was registered on ClinicalTrials.gov (Identifier Number: NCT04317911). Written informed consent was obtained prior to participation. Details, inclusion criteria, patient characteristics and further methods were described previously [111]. Briefly, 102 patients, admitted for atrial fibrillation cryoablation having an EF > 50%, were enrolled prospectively and screened for HFpEF by echocardiography, stress echocardiography, 6-min-walking-test and blood biomarker tests. 20 patients fulfilling the current HFpEF diagnosis criteria of the Heart Failure Association (HFA) of the ESC [72] were compared with 20 propensity score-matched controls derived from the enrolled non-HFpEF atrial fibrillation patients. Clinical follow-up was performed after 6 and 12 months including baseline examinations and 24 h holter ECG.

### Human ANGPTL4 plasma ELISA

Peripheral venous blood was drawn from the right femoral veins during vessel access prior to percutaneous pulmonary vein isolation and plasma aliquots stored at -80 °C. A human ANGPTL4 solid-phase sandwich ELISA kit (#EHANGPTL4, ThermoFisher) was used according to the provided manufacturer’s protocol. Samples were analyzed in doublets, 100 µL of 1:2 diluted plasma was incubated overnight and under agitation at 4 °C. Absorbance was detected at 450 nm using an EnSpire (PerkinElmer Inc) plate reader. The standard curve and final protein concentrations were calculated using Prism 9 (GraphPad). ROUT test for outlier identification was performed (Q = 0.1%) and one outlier in the HFpEF group was excluded, which exhibited a lipemic plasma sample. We tested our main hypothesis for difference in means between HFpEF and CT patients via Mann-Whitney U-test. Additional clinical covariates were tested for exploratory purpose and highest absolute correlation coefficients were reported. *P*-values were not adjusted in this second exploratory setting under the assumption of non-independence of clinical covariates.

## Supplementary Information

Below is the link to the electronic supplementary material

Supplementary file1 (DOCX 4882 KB)

## Data Availability

Code that is used in the analysis is accessible under https://github.com/saezlab/scell_hfpef. Single-cell data is available at the Gene Expression Omnibus (GSE275031). Other datasets used in this study are available online through referenced publications (MI study, E-MTAB-7895; AngII study, E-MTAB-8810).
